# Conserved expression of natural antisense transcripts in mammals

**DOI:** 10.1186/1471-2164-14-243

**Published:** 2013-04-12

**Authors:** Maurice HT Ling, Yuguang Ban, Hongxiu Wen, San Ming Wang, Steven X Ge

**Affiliations:** 1 Department of Mathematics and Statistics, South Dakota State University, Brookings, South Dakota SD 57007, USA; 2 Department of Genetics, Cell Biology and Anatomy, University of Nebraska Medical Center, Omaha, Nebraska NE 68198, United States of America

## Abstract

**Background:**

Recent studies had found thousands of natural antisense transcripts originating from the same genomic loci of protein coding genes but from the opposite strand. It is unclear whether the majority of antisense transcripts are functional or merely transcriptional noise.

**Results:**

Using the Affymetrix Exon array with a modified cDNA synthesis protocol that enables genome-wide detection of antisense transcription, we conducted large-scale expression analysis of antisense transcripts in nine corresponding tissues from human, mouse and rat. We detected thousands of antisense transcripts, some of which show tissue-specific expression that could be subjected to further study for their potential function in the corresponding tissues/organs. The expression patterns of many antisense transcripts are conserved across species, suggesting selective pressure on these transcripts. When compared to protein-coding genes, antisense transcripts show a lesser degree of expression conservation. We also found a positive correlation between the sense and antisense expression across tissues.

**Conclusion:**

Our results suggest that natural antisense transcripts are subjected to selective pressure but to a lesser degree compared to sense transcripts in mammals.

## Background

Recent studies suggest that a substantial portion of mammalian genomes are transcribed as non-coding RNA [1-4], including *cis*-natural antisense transcripts (*cis*-NATs) [5-8]. *Cis*-NATs are transcribed from the antisense counterpart of protein coding sequences, which may result in post-transcriptional gene silencing [9]. However, the extent to which *cis*-NATs are biologically functional and actively regulated remains a subject of debate [10]. Some studies had suggested that *cis*-NATs represent transcriptional noise [11,12], while others had reported supportive evidence for the function of various *cis*-NATs [13-16], especially in RNA editing [17], stability [18], and translation [19]. Growing evidence implicating a role of NATs in medical conditions, such as hypertension [20] and immune disorders [21], suggests a functional role for *cis*-NATs. However, it cannot be assumed that *cis*-NATs are as actively regulated as its sense counterparts.

Through our previous study, we developed an Antisense Transcriptome analysis using Exon array (ATE) approach for high-throughput expression analysis of NATs by using commercial oligonucleotide DNA microarrays [22]. The Affymetrix Exon array is an inexpensive high-density oligonucleotide microarray that has two unique features: (1) it has multiple probes for each of known or predicted exons, and (2) its signals are strand-specific because of the generation and labeling of single-stranded DNA targets. By modifying the recommended cDNA synthesis protocol, we demonstrated that it is possible to label targets in reverse orientation as what would be labeled according to the standard protocol (See Additional file 1: Figure S1). Thus, the cDNAs from known genes can no longer hybridize with these probes. Instead, any true hybridization signal must come from transcripts on the opposite strand, i.e., *cis*-NATs. Our preliminary microarray data on human Jurkat cells showed that the modified protocol can successfully detect a large number of NATs transcribed from known exonic loci [22]. Although limited to exonic NATs, using the Affymetrix exon arrays with our modified protocol provides a cost-efficient method to study the expression of NATs on ~ 1 million exonic loci.

The expression patterns of protein-coding genes [23,24] and orthologous genes that are essential to the organism [25] are evolutionarily conserved. Hence, it can be implied that orthologous transcripts that demonstrates expression conservation are likely to be biologically functional. Expression divergence of randomly assigned pairs of genes, by means of permutation, had been used as a baseline to approximate a neutral evolution of gene expression [23]. If orthologous cis-NATs show more correlated expression patterns when compared to randomly permuted cis-NAT pairs, it would provide evidence that cis-NATs are actively regulated or subject to selective pressure.

In this study, we measured the expression of antisense transcript across human, mouse, and rat using the ATE procedure [22]. Coupled with expression analysis of sense transcripts in the same samples, this will define a “double stranded” expression profile at the exon level. We report significant differences in expression divergence between antisense orthologous transcripts when compared to permuted pairs. However, the expression divergence of sense transcripts is significantly lower than that of antisense transcripts, suggesting that *cis*-NATs are subjected to selective pressure but to a lesser degree compared to sense transcripts.

## Methods

### Microarray data

Total RNA were purchased from Ambion (Austin, TX, USA). The RNA samples consist of human colon, mouse embryo, rat embryo, and 9 orthologous tissues from all 3 organisms, namely, brain, heart, kidney, liver, lung, spleen, ovary, testes, and thymus. Sense and antisense expression was measured using Affymetrix Exon 1.0 ST array according to manufacturer’s protocol and previously described modification for measuring antisense expression [22] respectively. Sense and antisense arrays were normalized separately using RMA method. The log expression values and exon annotations for core exon set were extracted using Affymetrix Expression Console. The final log expression values were averaged from two replicates.

### Defining orthology

Orthologous genes between human, rat, and mouse were identified from NCBI HomoloGene, build 65. The protein accession numbers were converted to gene accession numbers [26] and mapped to the microarray annotations for gene-level orthology. For exon-level orthology, three sets of exon sequences; human, mouse, and rat; were downloaded from USCS Genome Browser and used to generate BLAST databases. The exon sequence sets were compared using *blastn* and exon pairs with global sequence identity of more than 80% were considered to be orthologous. In event where one exon was found to have global sequence identity of more than 80% with more than one orthologous exons in the same organism, the exon with the highest global sequence identity were considered to be orthologous. In addition, exons with overlapping RefSeq transcripts on both sense and antisense strands were removed.

### Permutation test for expression divergence

Permutation test was used to evaluate whether the expression divergence of orthologous exons are statistically different from random. Expression divergence between 2 probesets is defined by Euclidean distance of the relative abundance, converted from microarray log expression value, using 9 orthologous tissues. At least one of the tissues must have expression higher than 6.5. Relative abundance is defined as the quotient of the microarray log expression value of the sample and the sum of the log expression values of the 9 tissues in the same set. Student’s t-test was used to test the expression divergence between the orthologous probesets and permuted pairs. Permuted pairs were generated by randomly assigning pairs of probesets from different organisms within the orthologous set. As a result, the number of orthologous pairs and permuted pairs are equal.

### Identifying tissue-specific NATs

Tissue-specific probesets were identified based on a previously described method [27] using 3 empirical criteria. Firstly, the log expression value of the highest expressing tissue must be higher than 6.5 which is the threshold for a detection p-value of 0.01 above background. Secondly, the Z-score of the log expression value for the highest expressing tissue must be higher than 2. Finally, the expression level of the highest expressing tissue must be at least one log higher than that of the second highest expressing tissue. Mouse and rat embryo samples were removed from the data set before identifying tissue-specific NAT probesets for the 9 orthologous tissues in rat and mouse.

### Identifying novel NATs

Novel NAT probesets were identified from the core Affymetrix exon probeset based on a previously described method [28] using BLASTN (version 2.2.25+). Each probeset was queried against RefSeq database (downloaded from NCBI on August 9, 2012) and EST database (downloaded from NCBI on May 15, 2012) for perfect matches. Query sequences without perfect matches in RefSeq database and EST database were considered to be novel. The strand option was set to “minus” to query only the reverse-complement of the query sequence (personal communication, Wayne Matten, NIH).

### Strand-specific RT-PCR

Strand-specific RT-PCR was used to validate the sense-antisense transcripts candidates. Total RNA samples were purchased from Clontech and tested for genomic DNA contamination by direct PCR at the cycling conditions of 95°C 30 seconds, 56°C 30 seconds, 72°C 45 seconds for 38 cycles before visualized in a 2% agarose gel. For RT-PCR, sense and antisense primers were designed for each candidate using Primer3. Strand-specific reverse transcription were performed for each candidate on the conditions: RNA (100 ng) were reverse-transcribed using M-MLV Reverse Transcriptase (Invitrogen) and gene-specific sense primer (for antisense detection) or antisense primer (for sense detection) (2 pmole) in 20 μl volume at 65°C for 5 minutes, 37°C for 50 minutes, 70°C for 15 minutes. 5 μl of cDNA was amplified in 50 μl containing sense primer (10 pmole), antisense primer (10 pmole), Taq polymerase (1.25 U, Promega), MgCl_2_ (1.5 mM) in ABI 9700 cycler under cycling conditions: 95°C for 7 minutes, 25 or 38 cycles of 95°C for 30 seconds, 56°C for 30 seconds, 72°C for 45 seconds, 72°C for 7 minutes, and visualized in 2% agarose gels.

## Results and discussion

Using the ATE protocol [22], we analyzed antisense transcription in 10 corresponding tissues for human, mouse and rat. The Affymetrix GeneChip Human Exon ST array includes 1.4 million probesets targeting exonic loci, of which 287,329 “core” probes are supported by full-length RNAs. Similar arrays for mouse and rat have 1.2 and 1 million probesets, and 231,465 and 92,751 core probesets, respectively. The same RNA sample and microarray were used to detect sense gene expression using the standard protocol. Two technical replicates were performed for each biological sample, resulting in a total of 120 hybridizations.

### Thousands of antisense transcripts are detected

67,649 (23.5%), 46,050 (19.9%), and 21,742 (23.4%) of human, mouse, and rat antisense exon probesets detected expression in at least one tissue, respectively. Of these, 33,313, 31,362, 14,180 antisense probesets do not overlap with RefSeq and EST sequences (Table 1), suggesting that these antisense transcripts are novel. This is similar to [28] who defined novelty as non-repetitive sequences that were not found in RefSeq database. The novel probeset list and sequences are given as Additional file 2: Dataset 1.

**Table 1:** Number of novel and tissue-specific antisense transcripts

	**Human**	**Mouse**	**Rat**
Total number of probesets supported by RefSeq annotations (Core probesets)	287,329 (100%)	231,465 (100%)	92,751 (100%)
Antisense transcripts that are not found in RefSeq database	278,827 (97.0%)	226,996 (98.1%)	91,648 (98.8%)
Antisense transcripts that are not found in EST database	140,496 (48.9%)	162,379 (70.2%)	66,943 (72.2%)
Antisense transcripts that are not found in both RefSeq or EST databases	139,918 (48.7%)	161,865 (69.9%)	66,865 (72.1%)
Antisense transcripts that are expressed in at least one tissue and not found in RefSeq or EST databases (novel antisense transcripts)	33,313 (11.6%)	31,362 (13.5%)	14,180 (15.3%)
Tissue-specific transcripts	14,485 (5.0%)	12,673 (5.5%)	11,358 (12.2%)
Tissue-specific transcripts and not found in RefSeq or EST database (novel, tissue-specific transcripts)	5,287 (1.8%)	7,652 (3.3%)	7,411 (8.0%)
Antisense transcripts that are expressed in at least one tissue and not found in RefSeq database (This is used for expression divergence analysis, Figure 5)	67,649 (23.5%)	46,050 (19.9%)	21,742 (23.4%)

To identify antisense transcripts with tissue-specific pattern of expression, we used empirical criteria similar to [27]. Our results suggest 14,485 (5.0% of core probesets) human antisense exon probesets, 12,673 (5.5% of core probesets) mouse antisense exon probesets and 11,358 (12.2% of core probesets) rat antisense exon probesets are tissue-specific. This may suggest a biological role for these antisense transcripts and warrant further investigation.

**Figure 1:**
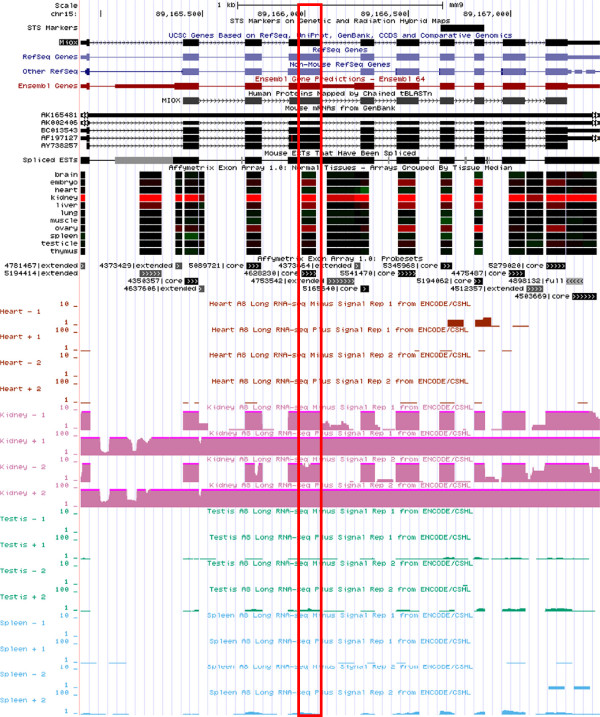
**Exon-level expression of mouse *Miox *gene.** USCS genome browser show that expression of Miox gene in mouse is kidney-specific for both Affymetrix exon array and RNA-seq [1,2]. The probeset in the box (Probeset ID 4628230) is an example of the probesets that shows kidney-specific expression at the antisense array, which is confirmed by RNA-seq.

**Figure 2:**
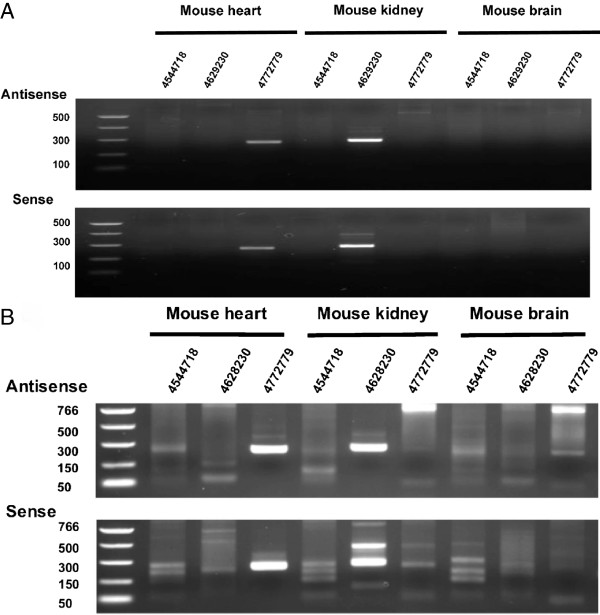
**Strand-specific PCR of 3 tissue-specific mouse probesets.** Panel **A** shows 25 cycles of amplification. Panel **B** showed 38 cycles of amplification. Probesets ID 4544718, 4628230, and 4772779 showed tissue-specific expressions to mouse embryo, mouse kidney, and mouse heart respectively. Probesets ID 4544718 targets the last exon of *myosin regulatory light chain 2, skeletal muscle* (*Mylpf*) gene. Probesets ID 4628230 targets the 4^th^ exon of *inositol oxygenase* (*Miox*) gene. Probesets ID 4772779 targets the 2^nd^ exon of *creatine kinase M-type* (*Ckm*) gene. Microarray expression data for these 3 probesets are given in Figure 3.

A number of the mouse tissue-specific antisense transcripts are confirmed using strand-specific RNA-seq data [29-31] in UCSC Genome Browser. As an example, the mouse probeset ID 4628230, corresponding to the 4^th^ exon of *inositol oxygenase* (*Miox*) gene, shows kidney-specific expression in both the Affymetrix Exon Array data and RNA-seq (Figure 1). Using strand-specific PCR targeting the 4^th^ exon of *Miox* gene, we confirmed the kidney-specific expression in both sense and antisense transcripts (Figure 2, Additional file 1: Figure S2). However, antisense expressions are much lower than sense expression and require larger quantities of RNA samples for antisense array [22]. This resulted in the need to normalize sense and antisense arrays separately. Therefore, it is difficult to quantify the ratio of sense to antisense expression on a global scale using microarray experiments. Inositol oxygenase had been reported to be involved in osmoregulation which accounts for its kidney-specificity [32].

**Figure 3:**
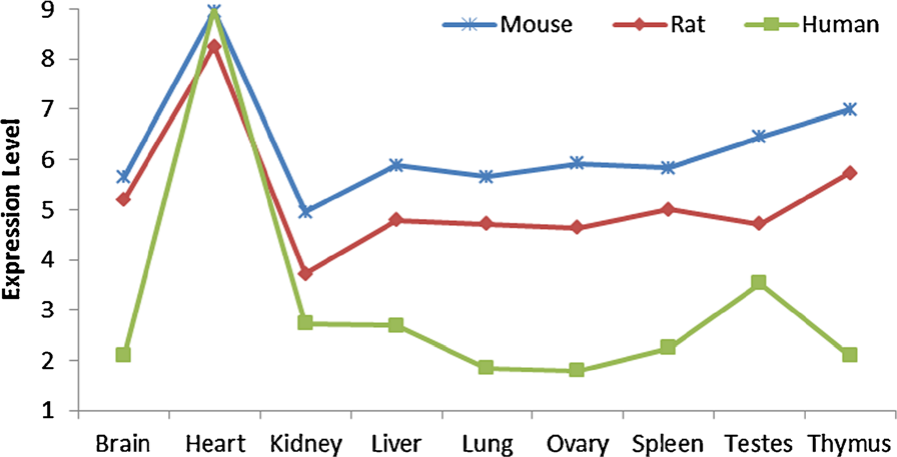
**Expression profile of an antisense heart-specific transcript (*creatine kinase M-type *gene) across all 3 species.** The mouse, rat, and human probesets are probesets ID 4772779, 5823307, and 3865287 respectively. The correlations between mouse/rat and mouse/human are 0.96 and 0.85 respectively, demonstrating expression conservation in all 3 species.

We choose 2 additional tissue-specific probes for strand-specific PCR verification, namely, probeset ID 4544718 which is embryo-specific, and probeset ID 4772779 which is heart-specific (Figure 3, Additional file 1: Figure S3 for primer design methodology). PCR from RNA samples without undergoing reverse transcription does not yield any visible bands (Additional file 1: Figure S4), which indicates no genomic DNA contamination in the RNA samples. Our results show that probeset ID 4772779 is only expressed in mouse heart and all 3 probes did not detect expression in mouse brain at the expected size. The PCR was performed with 25 and 38 extension cycles; hence, the lack of a strong band acts as strong support for lack of expression. Thus, experimental validation using strand-specific PCR supports the validity of our empirical criteria for the selection of tissue-specific antisense expression.

We had identified an example of human long noncoding RNA expression (Additional file 1: Figure S5) identified in NONCODE [33], which forms a sense-antisense pair at the 5^′^-end of *Ubiquitin-specific protease 25* (*USP25*). USP25 is involved in degrading mis-folded proteins in the endoplasmic reticulum [34]. This suggests that the expression of USP25 may be modulated by antisense transcript.

### Further validation of antisense transcript detection protocol

The average expression of antisense transcripts is lower than that of sense transcripts at both the gene and exon level (Additional file 1: Figures S6-S11). This is consistent with that of [22] who reported that the hybridization control probes were higher in antisense arrays, suggesting that the actual expression levels of transcripts on the antisense arrays were lower than sense arrays. The proportion of core exon probesets detected above background (DABG) with a detection p-value less than 0.01 (Additional file 1: Figure S12) were consistent with previous report [22]. Our results also suggest that probesets with DABG p-value of less than 0.01 have an intensity of at least 6.5.

Two technical replicates were performed for each tissue and our results show that core probeset intensities of the technical replicates are strongly correlated (0.86 < r < 0.99) in both sense and antisense arrays (Additional file 1: Figures S13-S24), suggesting that the experimental procedure is reproducible. The average intensity values from the two technical replicates were used for analysis.

For further validation of ATE procedure, we examined pre-designed probes for known antisense transcripts. We searched for extended probesets at the same genic location to core probesets but are located on the opposite strand. Some antisense transcripts should be detected by these extended probesets in the sense arrays, as well as the core probesets in the antisense array. We found positive correlation (0.50 < r < 0.58) between the expression level of core probesets in antisense array and the overlapping extended probeset in sense array (Additional file 1: Figure S25, S26). This is expected as the extended probesets in the sense array and the sense probesets in the antisense array are detecting the same antisense transcript.

### Sense gene expression is conserved as previously reported

**Figure 4:**
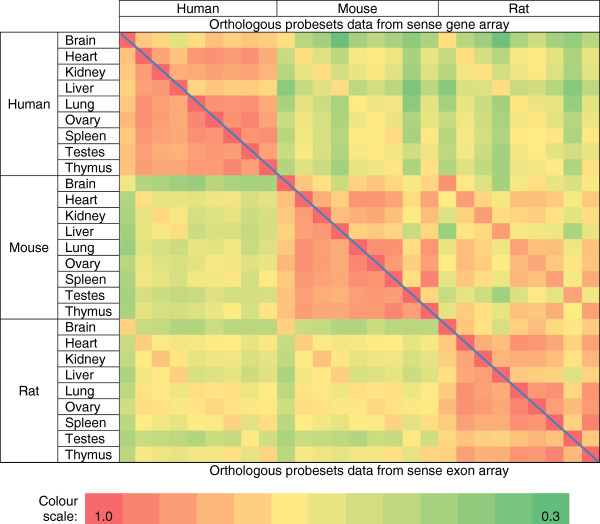
**Heatmaps of orthologous sense exon and gene probesets.** The colours represent the Pearson’s correlation coefficient of the orthologous probesets across any 2 tissues. The correlations for orthologous exons were calculated from exons probesets that are present across all 3 species based on 80% DNA identity. The correlations for orthologous genes were calculated from gene probesets that are present across all 3 species based on NCBI HomoloGene build 65. Pearson’s correlation ranged from 0.3 (green) to 1.0 (orange). The lower and upper triangles are heatmaps using data from exon and gene probesets respectively.

**Figure 5:**
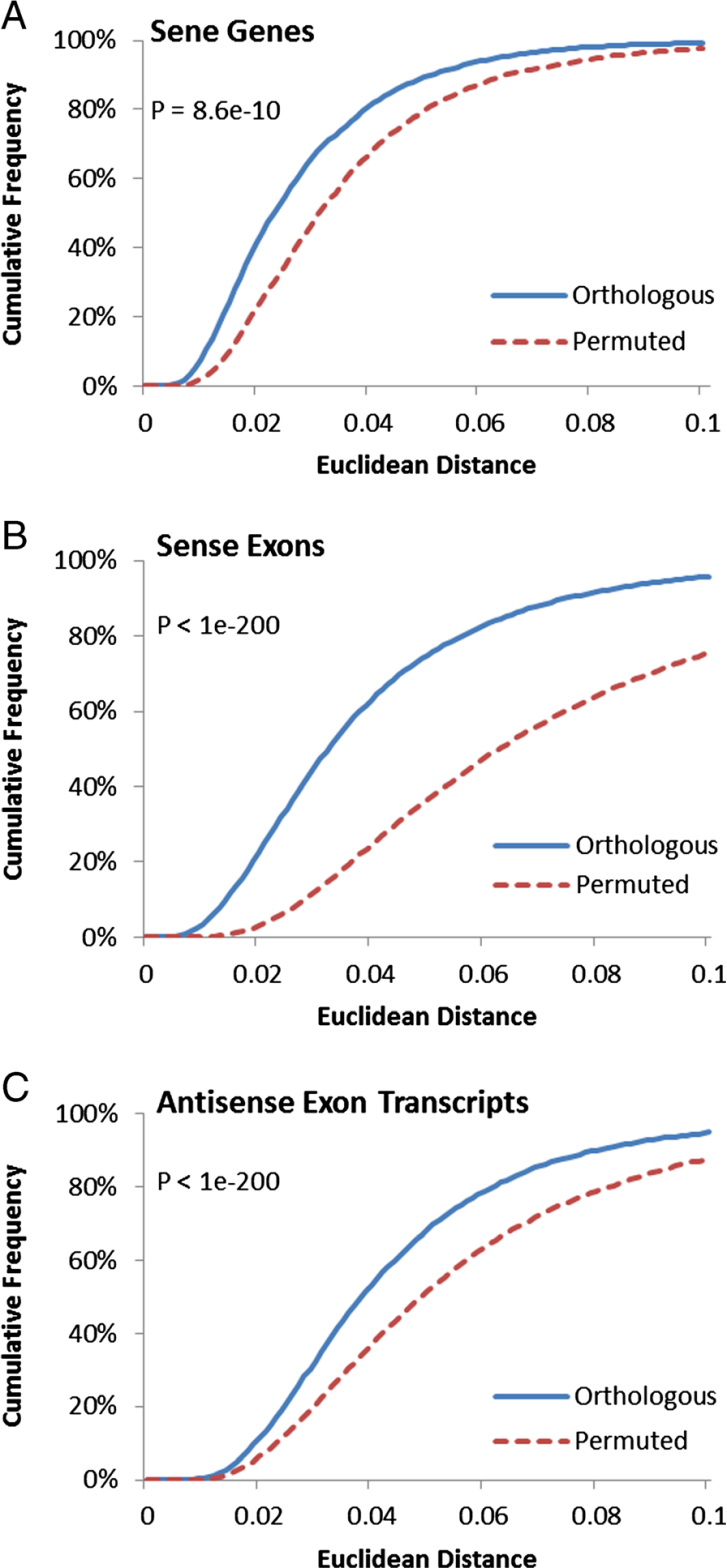
**Cumulative frequency of mouse/rat orthologous transcripts at gene and exon level against permuted pairs.** Vertical axis represents cumulative frequency. Horizontal axis represents expression divergence, calculated using Euclidean distance. Panel **A** shows the expression divergence distribution of protein-coding genes as defined by NCBI HomoloGene build 65 and its permuted pairs. Panel **B** shows the orthologous sense exons. Panel **C** shows antisense exon transcripts at the exon level excluding exons with antisense RefSeq transcripts. Probability density plots are given in Additional file 1: Figure S33.

To quantify expression divergence, we used Euclidean distance to measure the dissimilarity between the expression patterns across multiple tissues [23,35]. The expression divergence between orthologous genes is significantly lower than permuted pairs (Figure 4, Figure 5A) and the difference is correlated to evolutionary time between the two species (Additional file 1: Figure S27). In addition, we found stronger correlation between expression profiles of orthologous tissues than non-orthologous tissues (Figure 4). This corroborates [23] and validates our experimental and analytical procedures. Our results showed poor correlation (r < 0.167; Additional file 1: Figure S28) between the 2 measures used to calculate expression divergence, namely, Euclidean distance and Pearson’s correlation. Nevertheless, our results are robust (Additional file 1: Figure S27) despite using different expression divergence measures (Euclidean distance and Pearson’s correlation) or different intensity thresholds (full set of orthologous genes without threshold, or intensity of at least one orthologous gene to be above 6.5).

We extended the same analysis to exon-level expression data. Despite using different intensity thresholds to calculate expression divergence (Figure 4, Figure 5B, and Additional file 1: Figure S30), our results show that the expression divergence between orthologous sense exon transcripts is significantly lower than permuted pairs. Our results consistently demonstrate a stronger correlation between expression profiles of orthologous tissues than non-orthologous tissues as shown by the diagonal lines representing orthologous tissues between 2 organisms on the heatmap (Figure 4). As an example, the Pearson’s correlation between mouse and rat kidney is 0.72 while the correlation between mouse kidney and human brain is 0.44 (Additional file 1: Figure S31). Thus, our results validated our experiment and suggested that our analytical procedures are suitable for studying expression divergence in exon transcripts.

### Antisense transcript expressions suggest selective pressure

The same method for analyzing sense expression divergence at the exon level was used to analyze antisense expression divergence. Expression signal detected by each exon-specific probeset on the antisense array was treated as one antisense transcript. Using BLAST, we identified 7,460, 5,836 and 18,346 orthologous antisense transcripts for human/mouse, human/rat and mouse/rat comparisons, respectively. Student’s t-test with unequal variance between the expression divergence of orthologous antisense transcripts and that of permuted pairs suggests that the expression divergence of orthologous antisense transcripts are significantly lower from their respective permuted pairs in all 3 comparisons (p-value < 10^-8^; Figure 5C; Additional file 1: Figures S32A, S33A, and S35). Using different intensity thresholds (full set of orthologous exons without threshold, intensity of at least one orthologous probeset to be above 6.5 which is the intensity threshold for DABG p-value of less than 0.01, or intensity of at least one orthologous probeset to be above 8) also showed that the expression divergence of orthologous exons for antisense transcripts are significantly lower. At the same time, the Pearson’s correlations of expression divergence between permuted pairs (Additional file 1: Figures S32B, and S33B) are close to zero which is similar to that reported by [36]. However, it had been suggested that Pearson’s correlation and Euclidean distance can produce different results [37] and a novel randomization procedure had been proposed recently [38]. Using the randomization procedure proposed by [38], we arrive at the same conclusion that the expression divergence of orthologous antisense transcripts are significantly lower than their respective permuted pairs regardless of intensity thresholds (Additional file 1: Figure S34). This suggests that our results are not artifacts due to the use of Euclidean distance, different randomization methods or intensity thresholds. Therefore, our results suggest that the expressions of antisense transcripts are under selective pressure and their expressions are evolutionarily conserved which in agreement with the roles of antisense transcripts proposed by a large number of recent studies across different species (reviewed in [39,40]).

Our results show that the average difference between the expression divergence of the sense orthologs and its permuted counterparts is larger than the average difference between that of the antisense orthologs and its permuted counterparts (Figures 5B and 5C). This may imply on the extent of selective pressure on antisense transcripts. Permuted pairs approximate a purely neutral evolution of gene expression without selective pressure [23]. Hence, the deviation from permuted pairs may approximate the strength of selective pressure on top of a neutral background. Therefore, our results suggest that antisense expression is subjected to less selective pressure compared to sense expression.

### Positive correlation between sense and antisense expression

**Figure 6:**
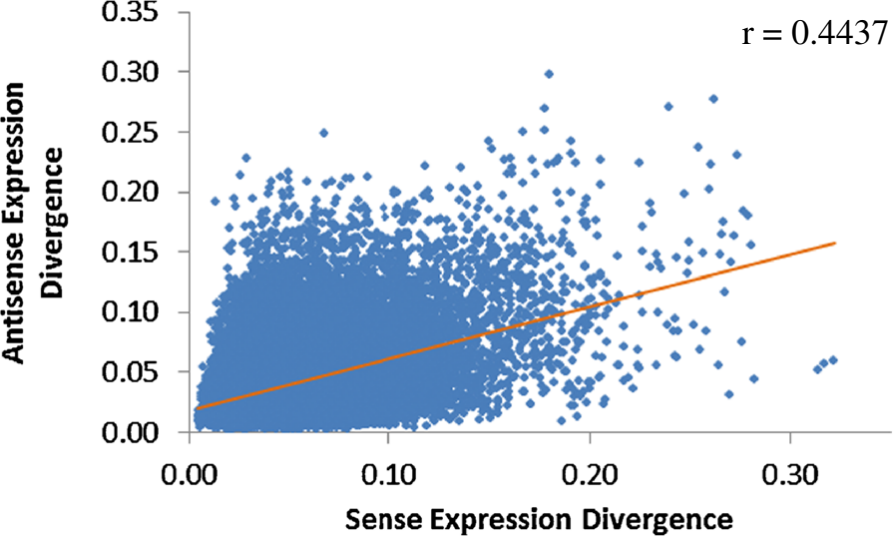
**Correlation between sense and antisense expression divergence.** The linear regression line is shown in orange.

Our results showed positive correlation between the sense and antisense expressions for all 30 tissues (Additional file 1: Figure S36-S39), demonstrating consistency across the 3 species. The Pearson’s correlation coefficient ranged from 0.333 (human brain) to 0.627 (rat thymus). Our result also showed positive correlation between sense and antisense divergence (Figure 6, r = 0.4437). This corroborates studies suggesting that sense and antisense expressions are positively correlated [36,41]. [36] reported that more sense/antisense pairs were positively correlated than negatively correlated, leading [36] to a conclusion that negative correlation between sense/antisense pairs are rare events. This is supported by [41] showing positive correlation between sense/antisense pairs in human breast epithelium and verified these findings with strand-specific quantitative PCR. However, our results corroborates [36], showing a large range of correlations between sense and antisense expressions. This may suggest a degree of independence between the regulation of sense and antisense expression. In addition, [36] reported that sense transcript detection occurred about 10 cycles earlier than antisense transcript detection using strand-specific quantitative PCR and [31] had reported lower antisense expression than their protein coding counterparts. This suggests even though both sense and antisense transcripts are present at the same time, there is a net abundance of sense transcripts.

Consistent positive correlation between sense and antisense divergence may suggest an over-arching regulatory mechanism for both sense and antisense expression while the large proportion of uncorrelated sense and antisense expression may suggest a layer of independent regulation. Chromatin structure had been shown to affect the accessibility of RNA polymerase II and other transcription factors to the site of transcription by means of methylation and acetylation [42,43]; thereby, playing an important factor in regulating gene expression. However, the role of chromatin structure in antisense transcript regulation had only been recently reported by [10] using chromatin immuno-precipitation and demonstrated positive correlation between *cis*-NAT promoter activity, the presence of RNA polymerase II histones modification and the resulting antisense RNA-seq read density, suggesting that chromatin structure also may be involved in *cis*-NAT transcription.

## Conclusion

In summary, we analyzed the expression of antisense transcripts in corresponding tissues across 3 species and found evidence for the conserved expression of these transcripts, similar to what have been observed in protein-coding genes. This supports the idea that expression of antisense transcripts is regulated and subject to selection pressure. Our result is based on a large number of antisense transcripts, supplementing previous studies of the functions of specific antisense transcripts. In addition, the tissue-specific expression pattern of some antisense transcripts might guide future in-depth study of their potential function.

One could argue that the conserved expression of natural antisense transcripts might be a by-product of the regulated and conserved expression of the corresponding protein-coding genes. Chromatin remodelling is one mechanism of gene regulation that makes the DNA sequences accessible to transcriptional protein complexes. It is possible for antisense transcripts to get a “free ride” when this happens in a regulated manner. Further studies are necessary to fully address these possibilities, especially using new technologies like strand-specific RNA-Seq.

## Competing interests

The authors declare that they have no competing interests.

## Authors’ contributions

ML performed the bioinformatic analysis and drafted the manuscript. YB contributed the randomization procedures and participated in bioinformatics analyses. HW validated the results using PCR. SMW and SXG conceived and oversaw this project. All authors read and approved the final manuscript.

## Supplementary Material

Additional file 1 Figures S1-S39, Supplementary methods and additional figures. Click here for file

Additional file 2 **Supplementary Dataset 1.** List of novel antisense transcripts, with associated sequences, identified in this study. Click here for file

## Data access

Microarray data for this work had been deposited in NCBI GEO as GSE41462 (antisense data) and GSE41464 (sense data). The sense and antisense expression data for various normal tissues in human, mouse and rat will also be accessed at UCSC Genome Browser as a custom track.
