# Supplementary material for: Conserved expression of natural antisense transcripts in mammals
Source: BMC Genomics. 2013 Apr 12;14:243. doi: 10.1186/1471-2164-14-243 (PMC3635984; doi:10.1186/1471-2164-14-243)
Supplement: Additional file 1 — Figures S1-S39, Supplementary methods and additional figures. [file 1471-2164-14-243-S1.pdf]

Supplementary Figures

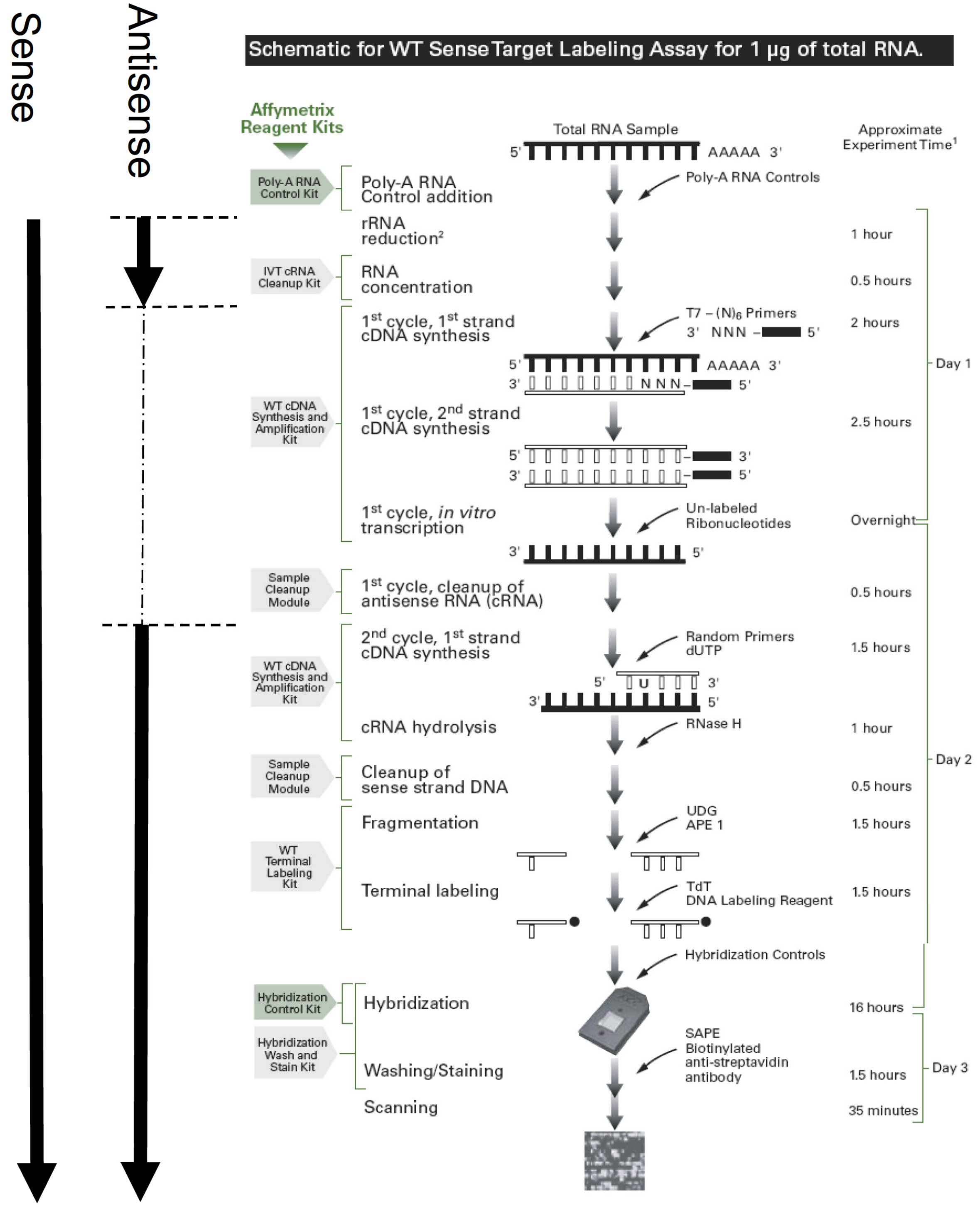

Figure S1. The modified protocol compared with standard Affymetrix protocol. In our antisense transcriptome analysis using Exon array (ATE) protocol, the first cycle cDNA synthesis and *in vitro* transcription is skipped. The image on the right is courtesy of Affymetrix, Inc.

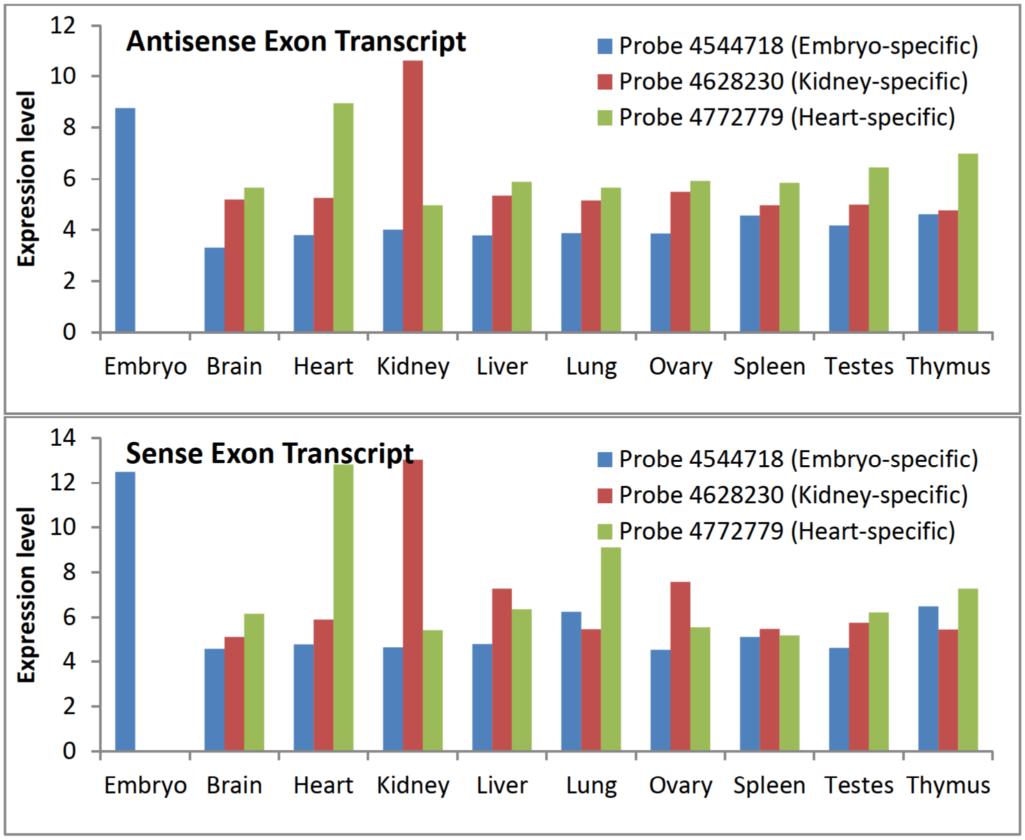

Figure S2. Expression data from microarray for probesets used in strand-specific PCR validation (Figure 4). All 3 probesets are from mouse exon array.

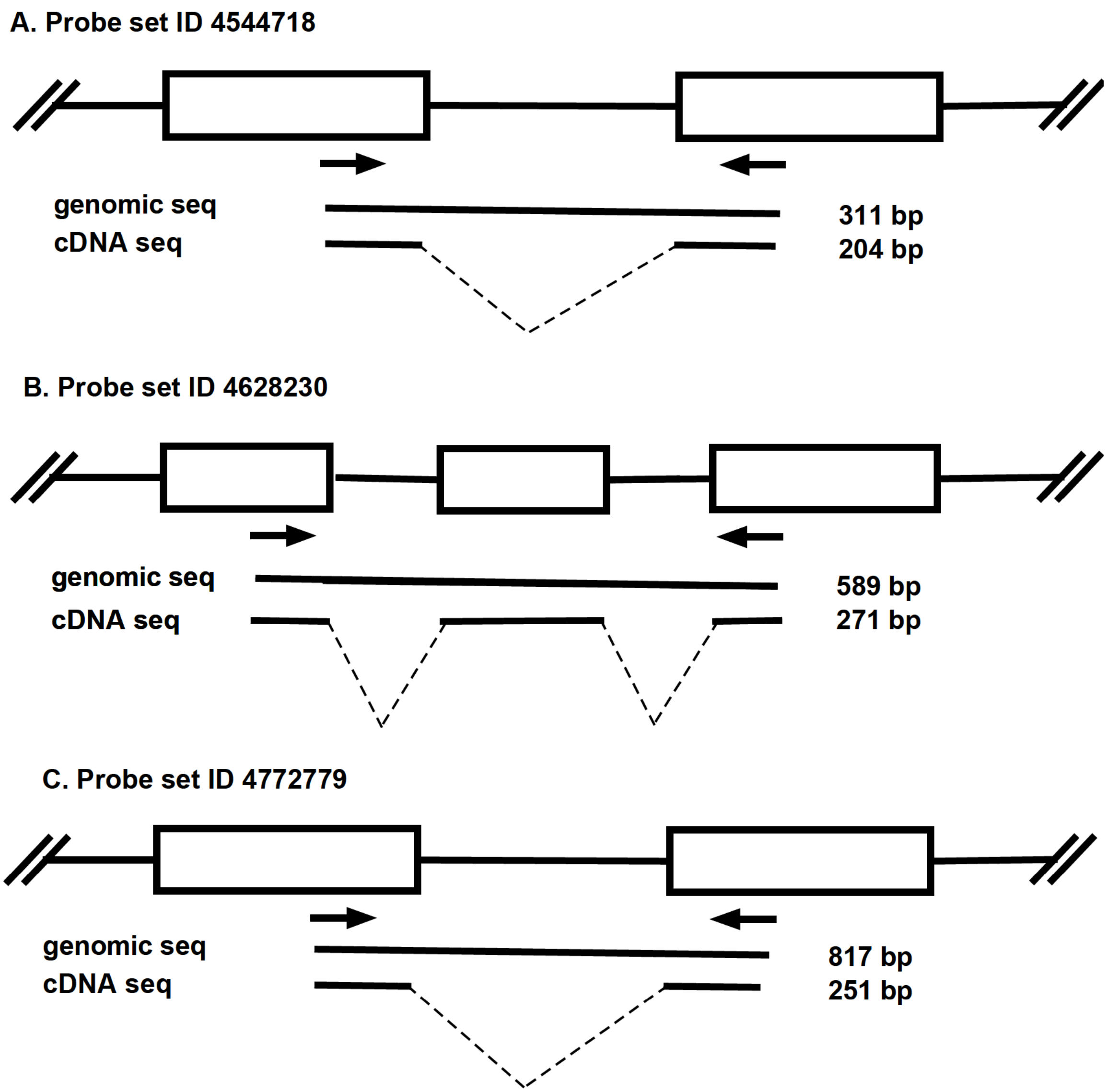

Figure S3. Primer design for strand-specific RT-PCR. For each targeted transcript, the corresponding probe sequence in array was used to map to the reference genome. Sense primer and antisense primer were designed in different exons across one intron (probe 4544718 and 4772779) or two introns/one exon (probe 4628230). Different size from mRNA and genomic DNA for each targeted sequence will determine if the RT-PCR products were from mRNA templates or from genomic DNA contamination. In fact, the sizes of strand-specific RT-PCR fit with the size of corresponding mRNA; therefore, rule out the possibility of genomic DNA contamination.

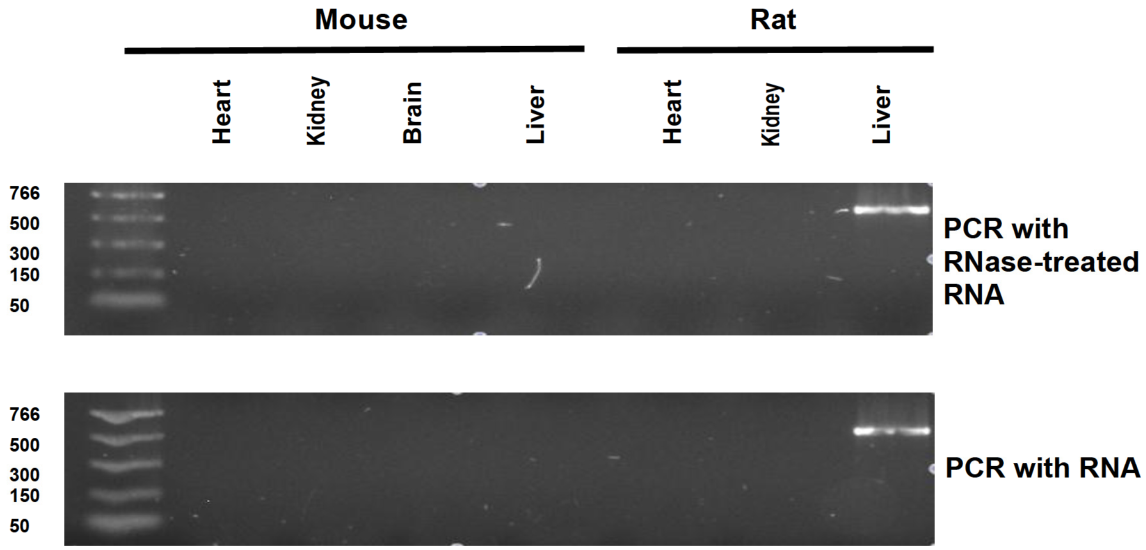

Figure S4. PCR of RNA samples. The issue of genomic DNA contamination: All the RNA samples used for the study were pre-tested for possible DNA contamination. The upper panel shows PCR result using RNase A-treated RNA samples as the templates, the lower panel shows PCR results using RNA directly as the templates. In both tests, all RNA samples show no sign of genomic DNA contamination except one (rat liver RNA), which was not used in validation study.

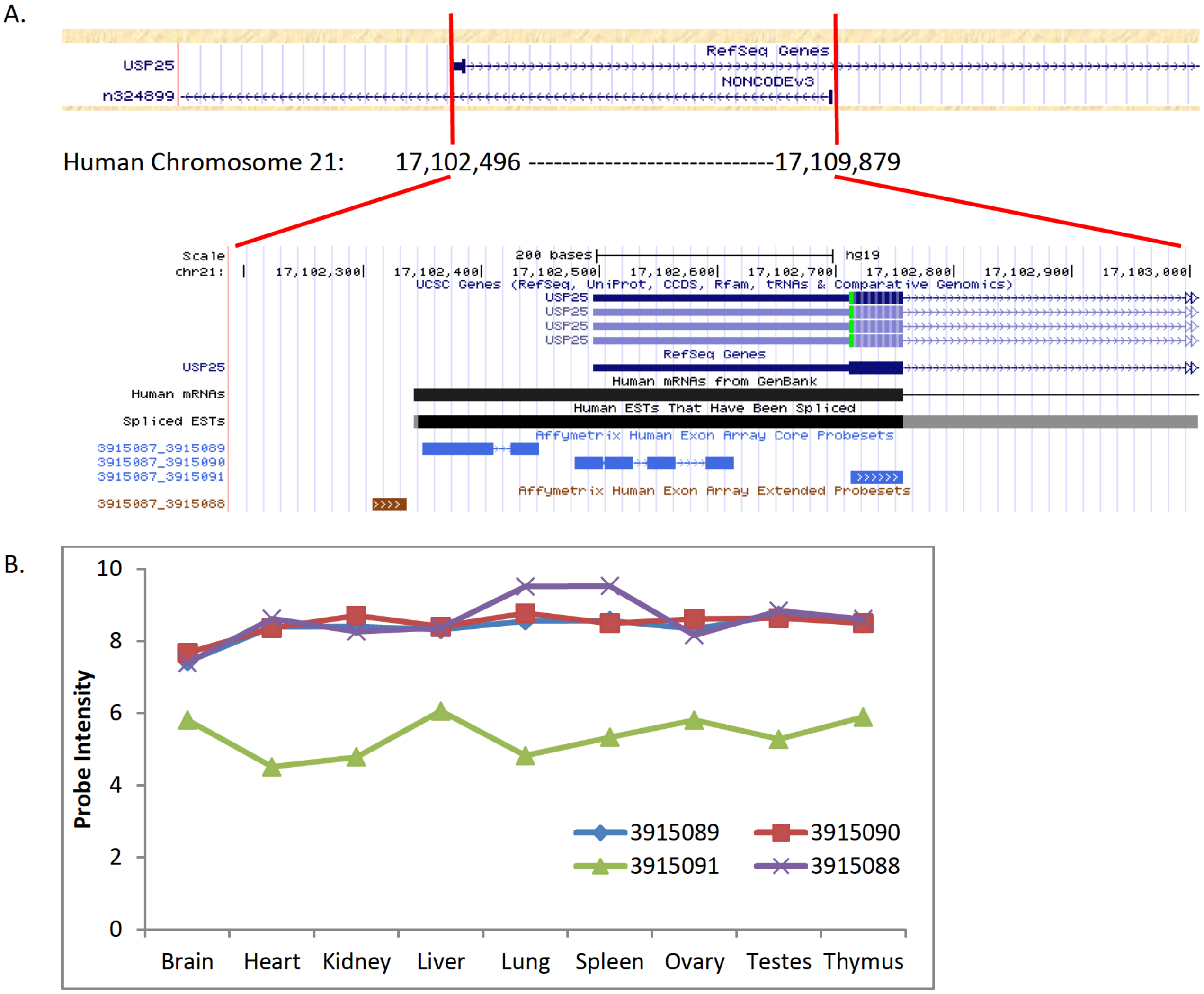

Figure S5. Expression profile of a human long noncoding RNA. Panel A show an overlapping long noncoding RNA identified in NONCODE version 3 (Bu et al., 2012), n324899, which overlaps with human USP25 in chromosome 21 and in the opposite orientation. The region of overlap is between 17,102,496 to 17,109,879 base-pairs, which contain 3 Affymetrix core probesets (probeset IDs 3915089, 3915090 and 3915091) and 1 extended probeset, probeset ID 3915088. Panel B shows the expression profile of the antisense transcript which corresponds to NONCODE transcript ID n324899. **Main Findings: Our antisense microarray results confirm the presence of expected NONCODE transcript.**

A.
B.

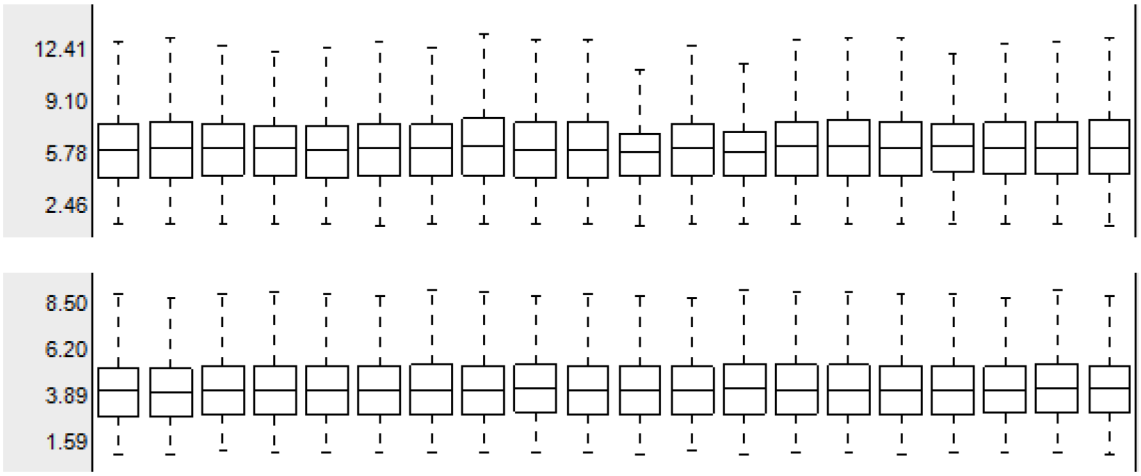

Figure S6. <u>Distribution of core probeset intensity after RMA normalization: human at gene probeset level.</u> Panel A shows intensity of sense gene probesets. Panel B shows intensity of antisense gene probesets. The y-axis represents the probeset intensities. The samples are (from left to right): brain replicate 1, brain replicate 2, colon replicate 1, colon replicate 2, heart replicate 1, heart replicate 2, kidney replicate 1, kidney replicate 2, liver replicate 1, liver replicate 2, lung replicate 1, lung replicate 2, ovary replicate 1, ovary replicate 2, spleen replicate 1, spleen replicate 2, testes replicate 1, testes replicate 2, thymus replicate 1, and thymus replicate 2.

A.
B.

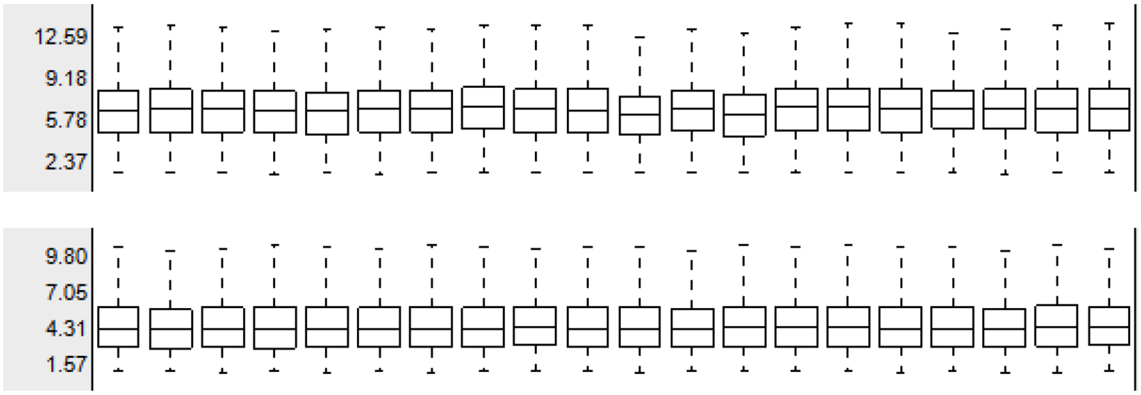

Figure S7. <u>Distribution of core probeset intensity after RMA normalization: human at exon probeset level.</u> Panel A shows intensity of sense exon probesets. Panel B shows intensity of antisense exon probesets. The y-axis represents the probeset intensities. The samples are (from left to right): brain replicate 1, brain replicate 2, colon replicate 1, colon replicate 2, heart replicate 1, heart replicate 2, kidney replicate 1, kidney replicate 2, liver replicate 1, liver replicate 2, lung replicate 1, lung replicate 2, ovary replicate 1, ovary replicate 2, spleen replicate 1, spleen replicate 2, testes replicate 1, testes replicate 2, thymus replicate 1, and thymus replicate 2.

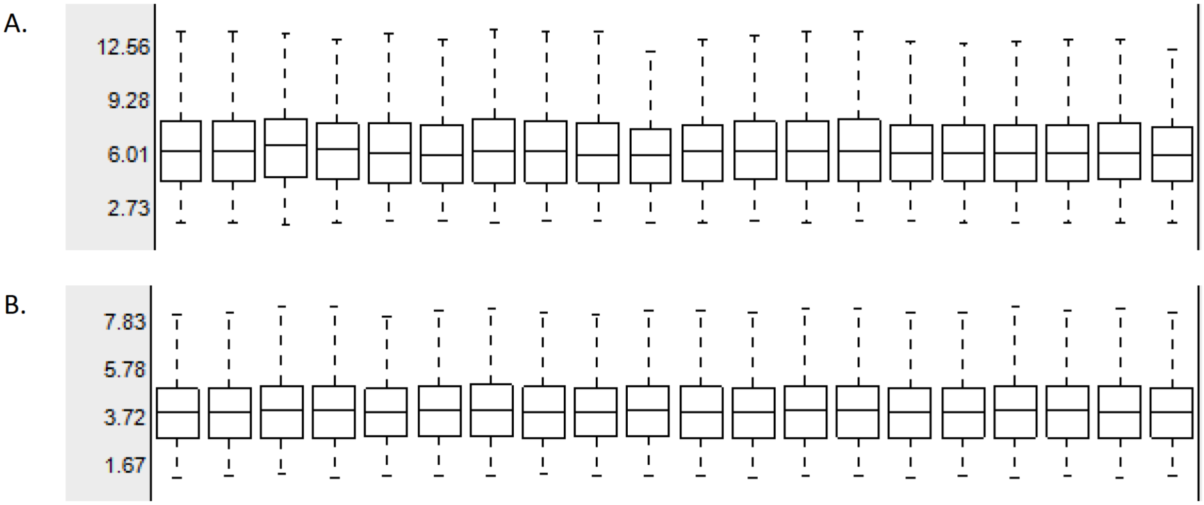

Figure S8. <u>Distribution of core probeset intensity after RMA normalization: mouse at gene probeset level.</u> Panel A shows intensity of sense gene probesets. Panel B shows intensity of antisense gene probesets. The y-axis represents the probeset intensities. The samples are (from left to right): brain replicate 1, brain replicate 2, embryo replicate 1, embryo replicate 2, heart replicate 1, heart replicate 2, kidney replicate 1, kidney replicate 2, liver replicate 1, liver replicate 2, lung replicate 1, lung replicate 2, ovary replicate 1, ovary replicate 2, spleen replicate 1, spleen replicate 2, testes replicate 1, testes replicate 2, thymus replicate 1, and thymus replicate 2.

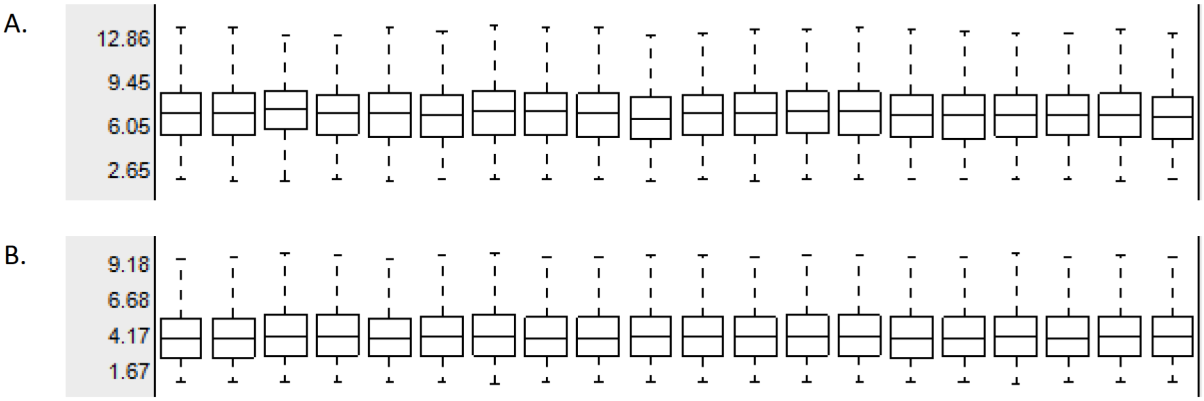

Figure S9. <u>Distribution of core probeset intensity after RMA normalization: mouse at exon probeset level.</u> Panel A shows intensity of sense exon probesets. Panel B shows intensity of antisense exon probesets. The y-axis represents the probeset intensities. The samples are (from left to right): brain replicate 1, brain replicate 2, embryo replicate 1, embryo replicate 2, heart replicate 1, heart replicate 2, kidney replicate 1, kidney replicate 2, liver replicate 1, liver replicate 2, lung replicate 1, lung replicate 2, ovary replicate 1, ovary replicate 2, spleen replicate 1, spleen replicate 2, testes replicate 1, testes replicate 2, thymus replicate 1, and thymus replicate 2.

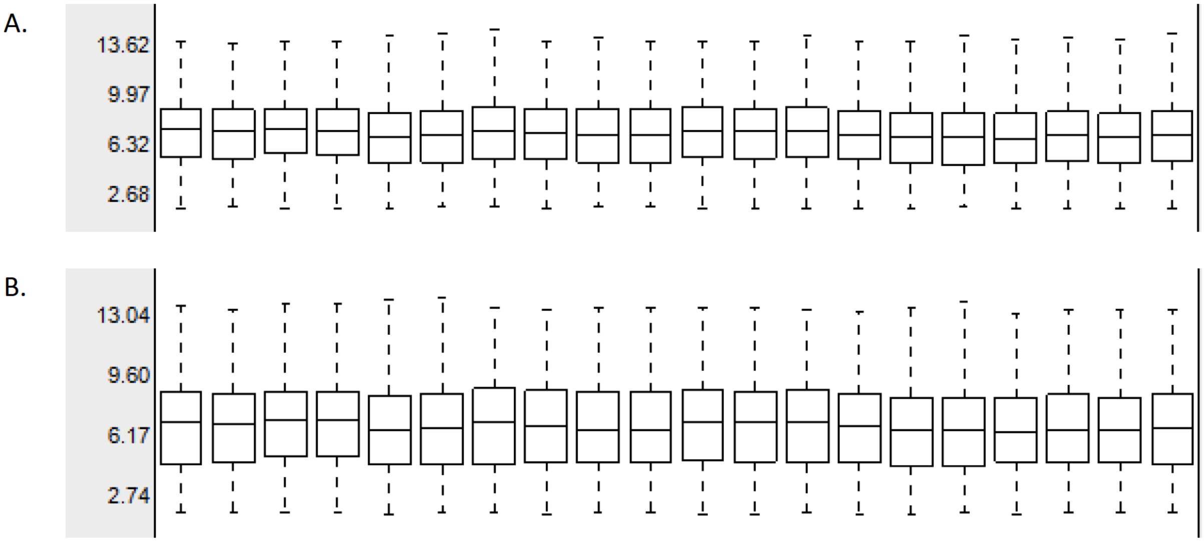

Figure S10. Distribution of core probeset intensity after RMA normalization: rat at gene probeset level. Panel A shows intensity of sense gene probesets. Panel B shows intensity of antisense gene probesets. The y-axis represents the probeset intensities. The samples are (from left to right): brain replicate 1, brain replicate 2, embryo replicate 1, embryo replicate 2, heart replicate 1, heart replicate 2, kidney replicate 1, kidney replicate 2, liver replicate 1, liver replicate 2, lung replicate 1, lung replicate 2, ovary replicate 1, ovary replicate 2, spleen replicate 1, spleen replicate 2, testes replicate 1, testes replicate 2, thymus replicate 1, and thymus replicate 2.

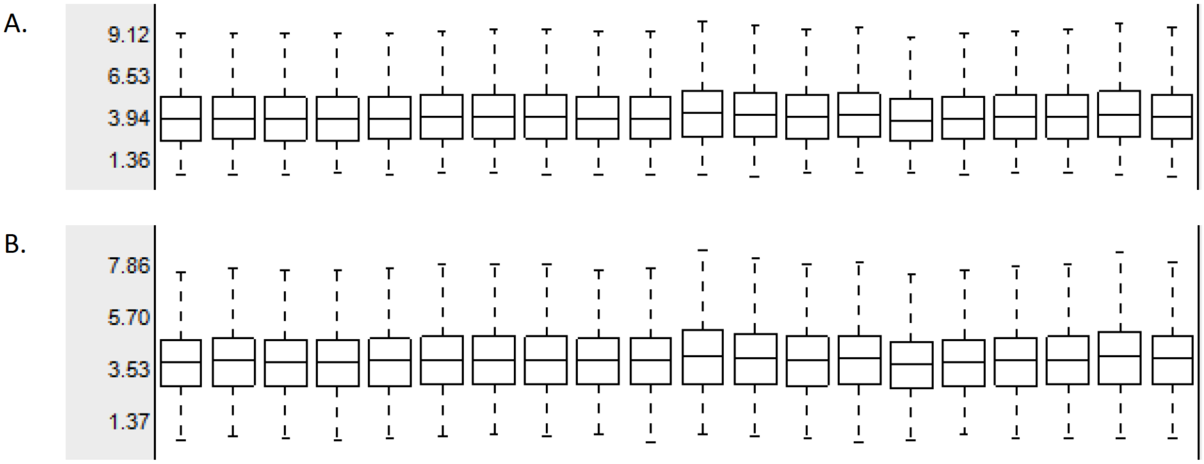

Figure S11. Distribution of core probeset intensity after RMA normalization: rat at exon probeset level. Panel A shows intensity of sense exon probesets. Panel B shows intensity of antisense exon probesets. The y-axis represents the probeset intensities. The samples are (from left to right): brain replicate 1, brain replicate 2, embryo replicate 1, embryo replicate 2, heart replicate 1, heart replicate 2, kidney replicate 1, kidney replicate 2, liver replicate 1, liver replicate 2, lung replicate 1, lung replicate 2, ovary replicate 1, ovary replicate 2, spleen replicate 1, spleen replicate 2, testes replicate 1, testes replicate 2, thymus replicate 1, and thymus replicate 2.

A.

B.

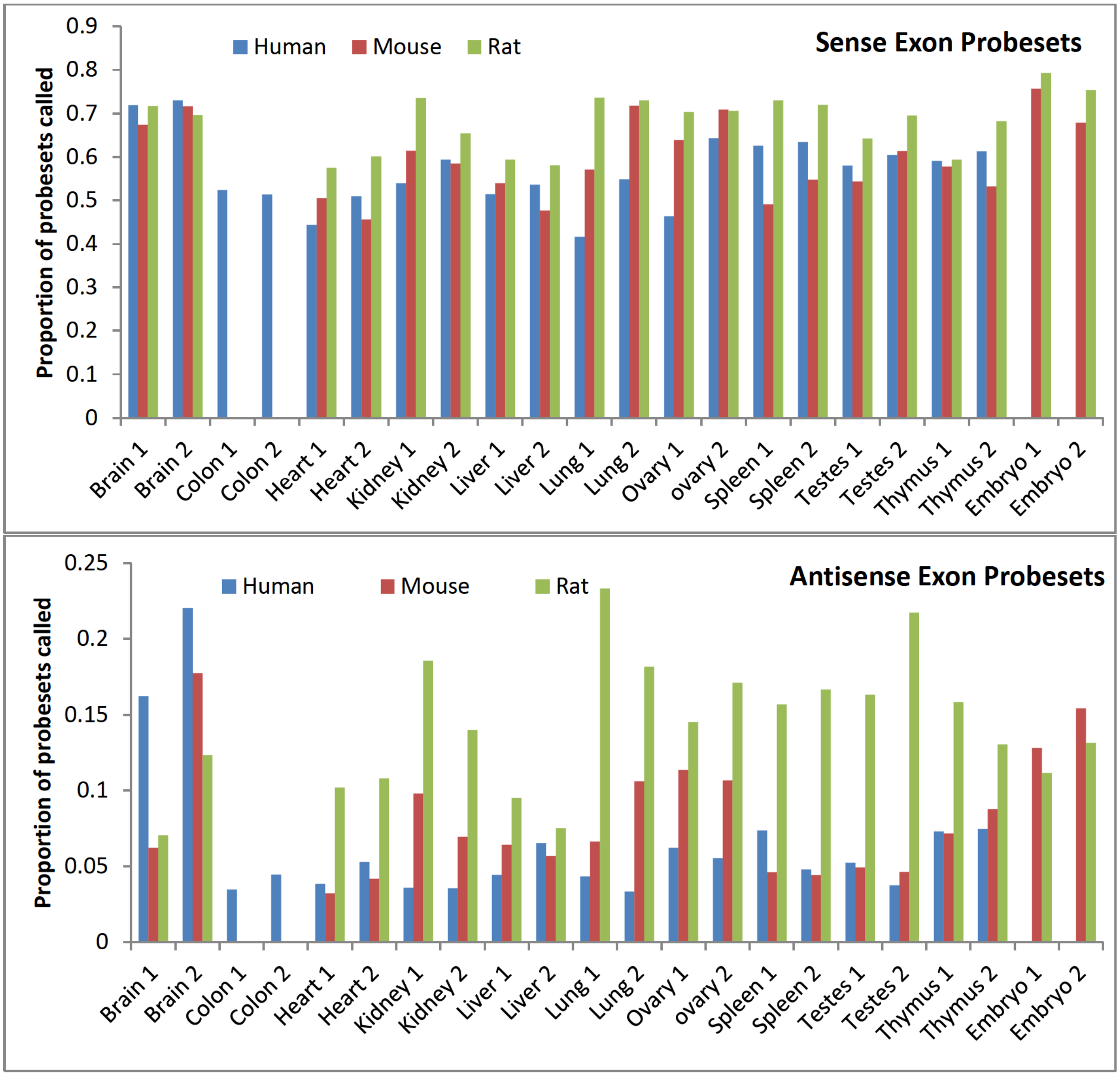

Figure S12. Proportion of exon-level probeset detected above background (DABG), with a detection p-value of < 0.01. Panel A shows sense arrays. Panel B shows antisense arrays. **Main Findings: The proportion of detected probesets in the sense and antisense exon arrays were 61.5% (standard deviation of 9.2%) and 9.6% (standard deviation of 5.5%) respectively, which is relatively consistent with Ge et al. (2008) reporting 41.4% sense detection and 13.4% antisense detection.**

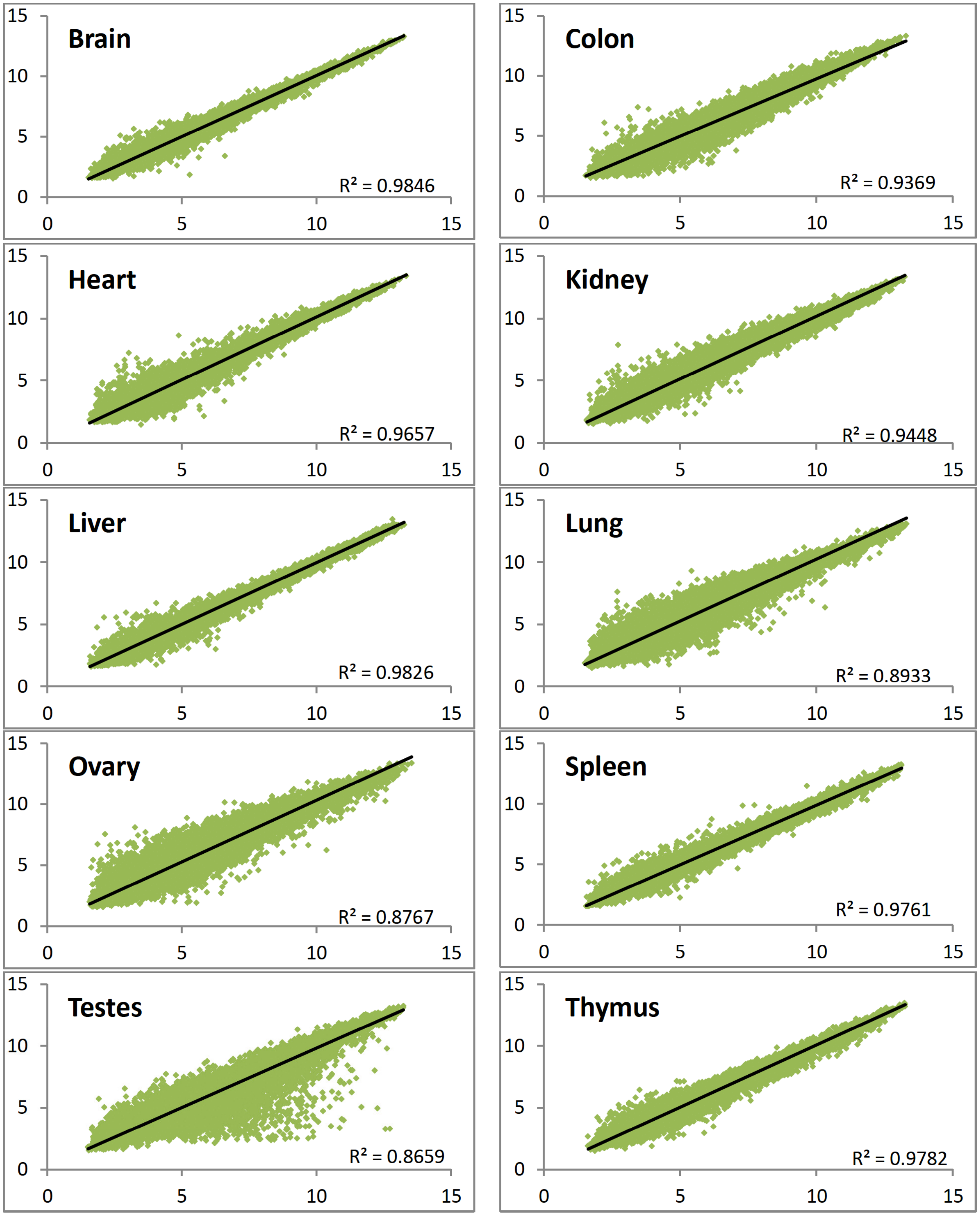

Figure S13. Correlation between intensity of technical replicates (n = 2) for human sense gene array. The y-axis and x-axis are the expression intensities (in logarithmic scale) of the first and second replicate respectively. **Main Findings: All technical replicates are correlated.**

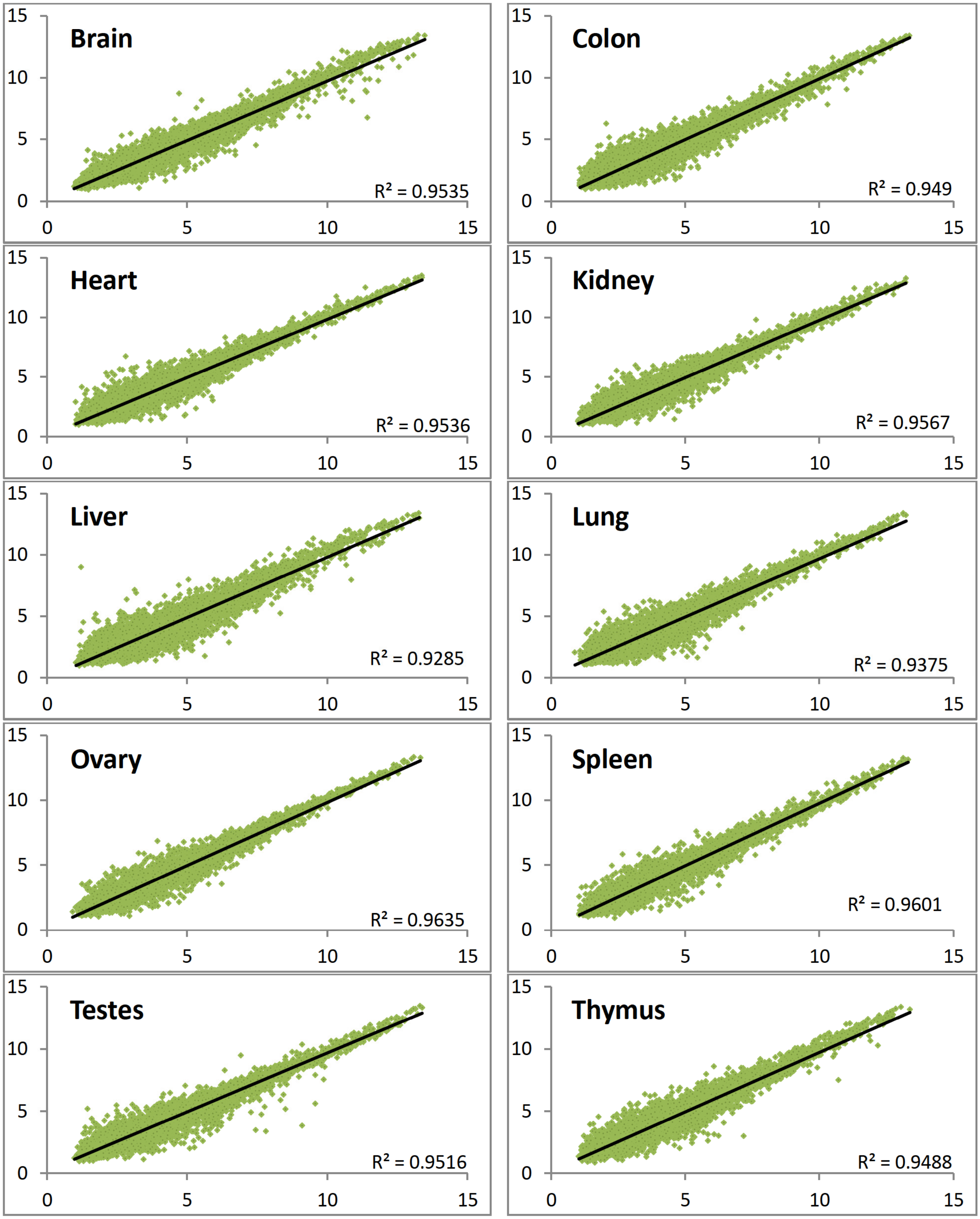

Figure S14. Correlation between intensity of technical replicates (n = 2) for human antisense gene array. The y-axis and x-axis are the expression intensities (in logarithmic scale) of the first and second replicate respectively. **Main Findings: All technical replicates are correlated.**

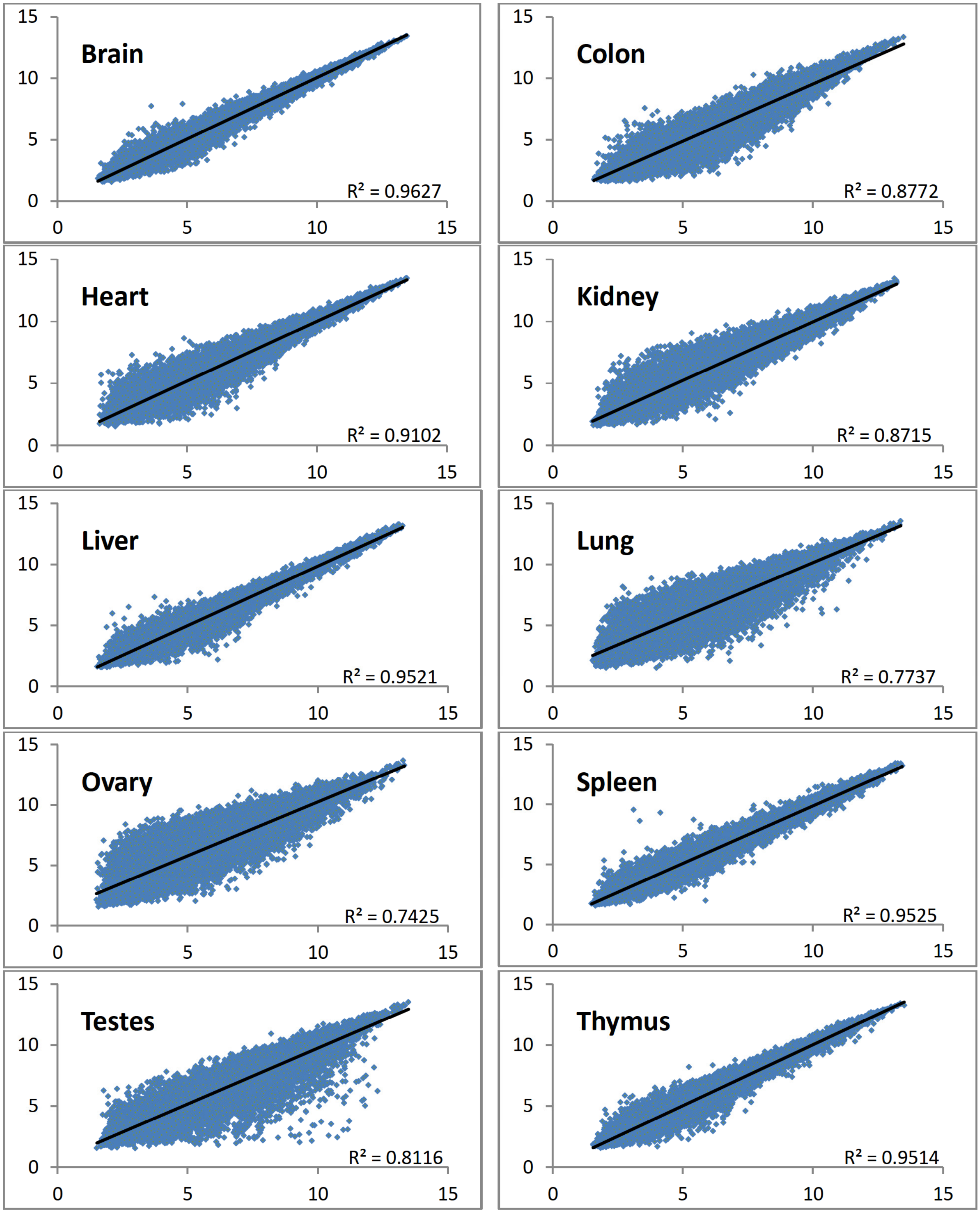

Figure S15. Correlation between intensity of technical replicates (n = 2) for human sense exon array. The y-axis and x-axis are the expression intensities (in logarithmic scale) of the first and second replicate respectively. **Main Findings: All technical replicates are correlated.**

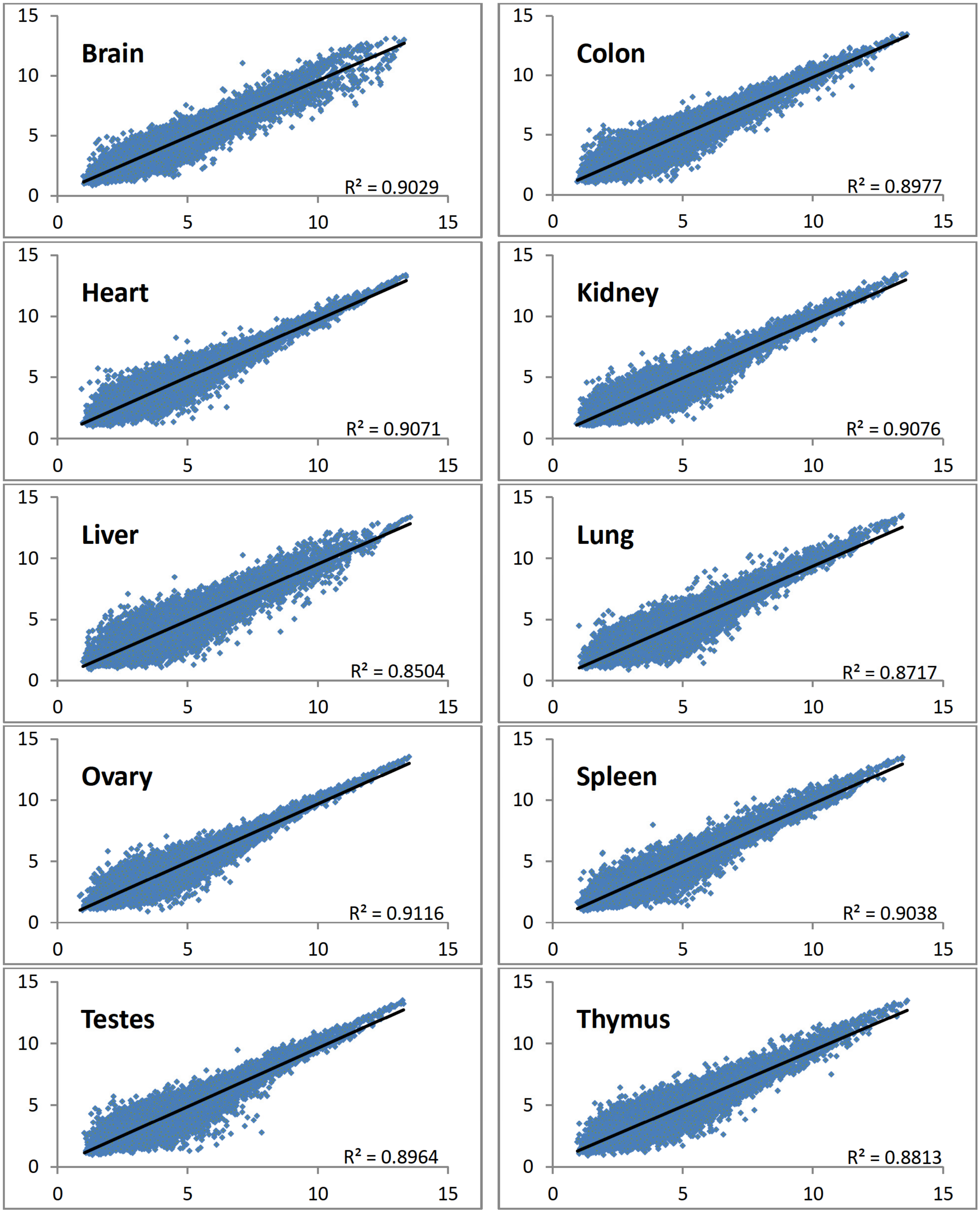

Figure S16. Correlation between intensity of technical replicates (n = 2) for human antisense exon array. The y-axis and x-axis are the expression intensities (in logarithmic scale) of the first and second replicate respectively. **Main Findings: All technical replicates are correlated.**

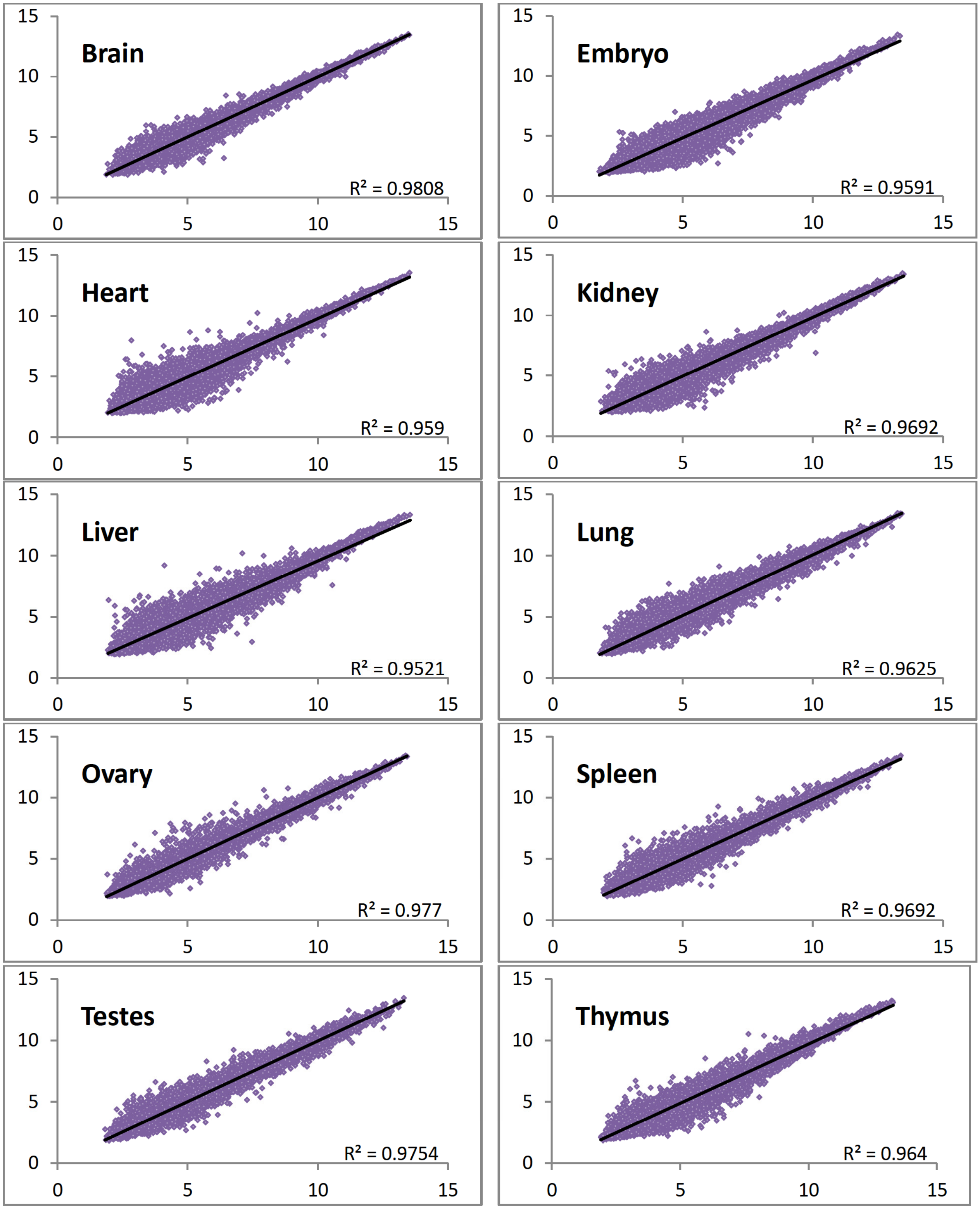

Figure S17. Correlation between intensity of technical replicates (n = 2) for mouse sense gene array. The y-axis and x-axis are the expression intensities (in logarithmic scale) of the first and second replicate respectively. **Main Findings: All technical replicates are correlated.**

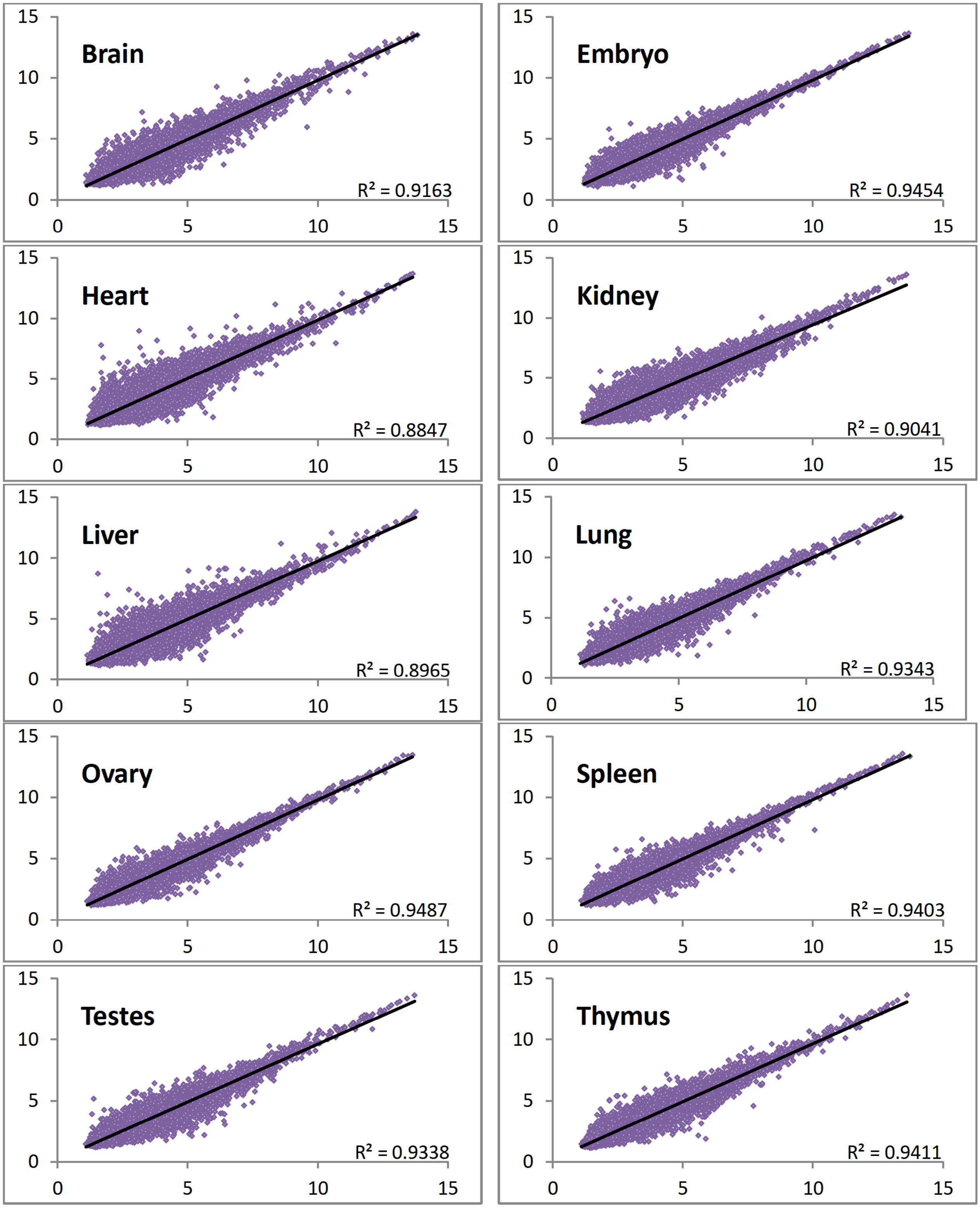

Figure S18. Correlation between intensity of technical replicates (n = 2) for mouse antisense gene array. The y-axis and x-axis are the expression intensities (in logarithmic scale) of the first and second replicate respectively. **Main Findings: All technical replicates are correlated.**

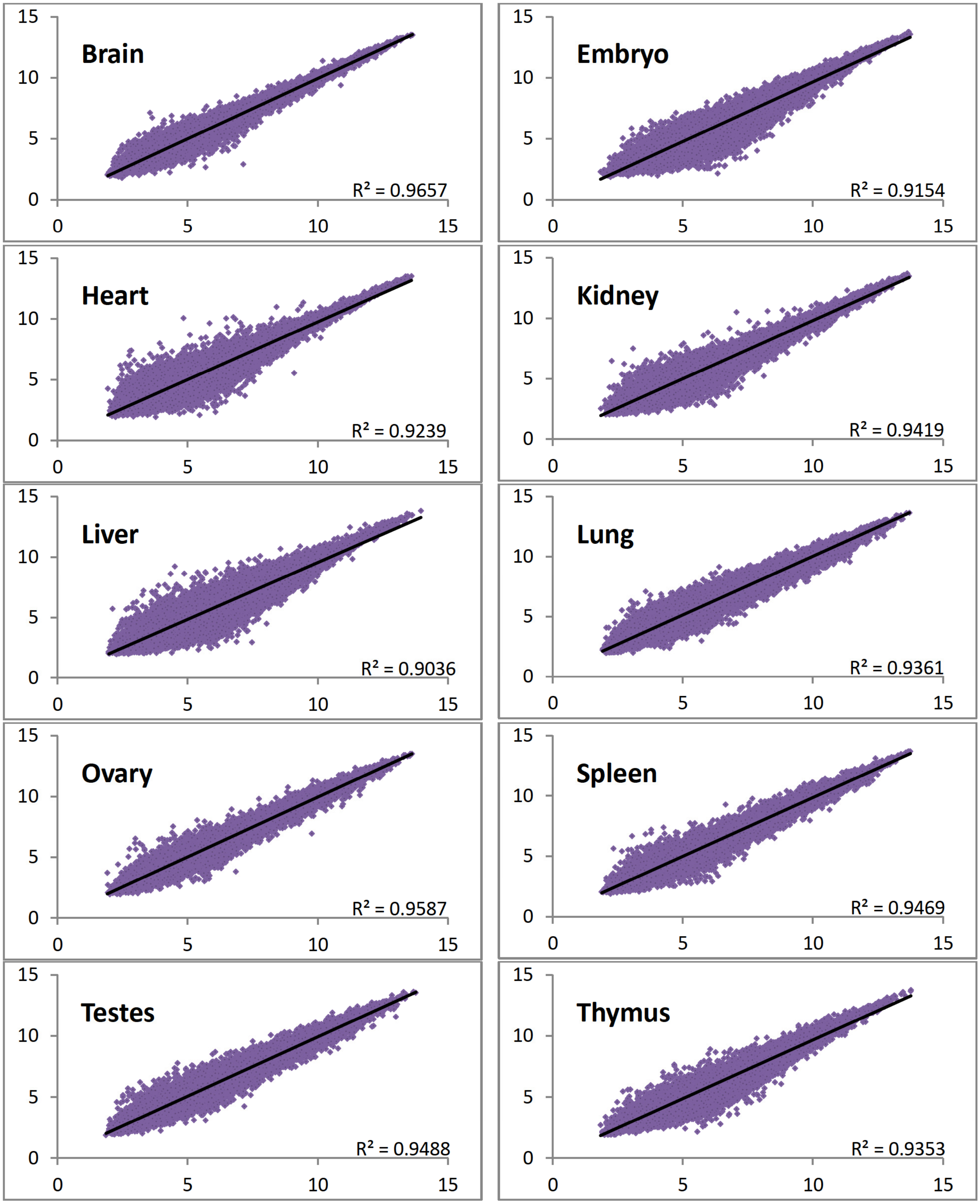

Figure S19. Correlation between intensity of technical replicates (n = 2) for mouse sense exon array. The y-axis and x-axis are the expression intensities (in logarithmic scale) of the first and second replicate respectively. **Main Findings: All technical replicates are correlated.**

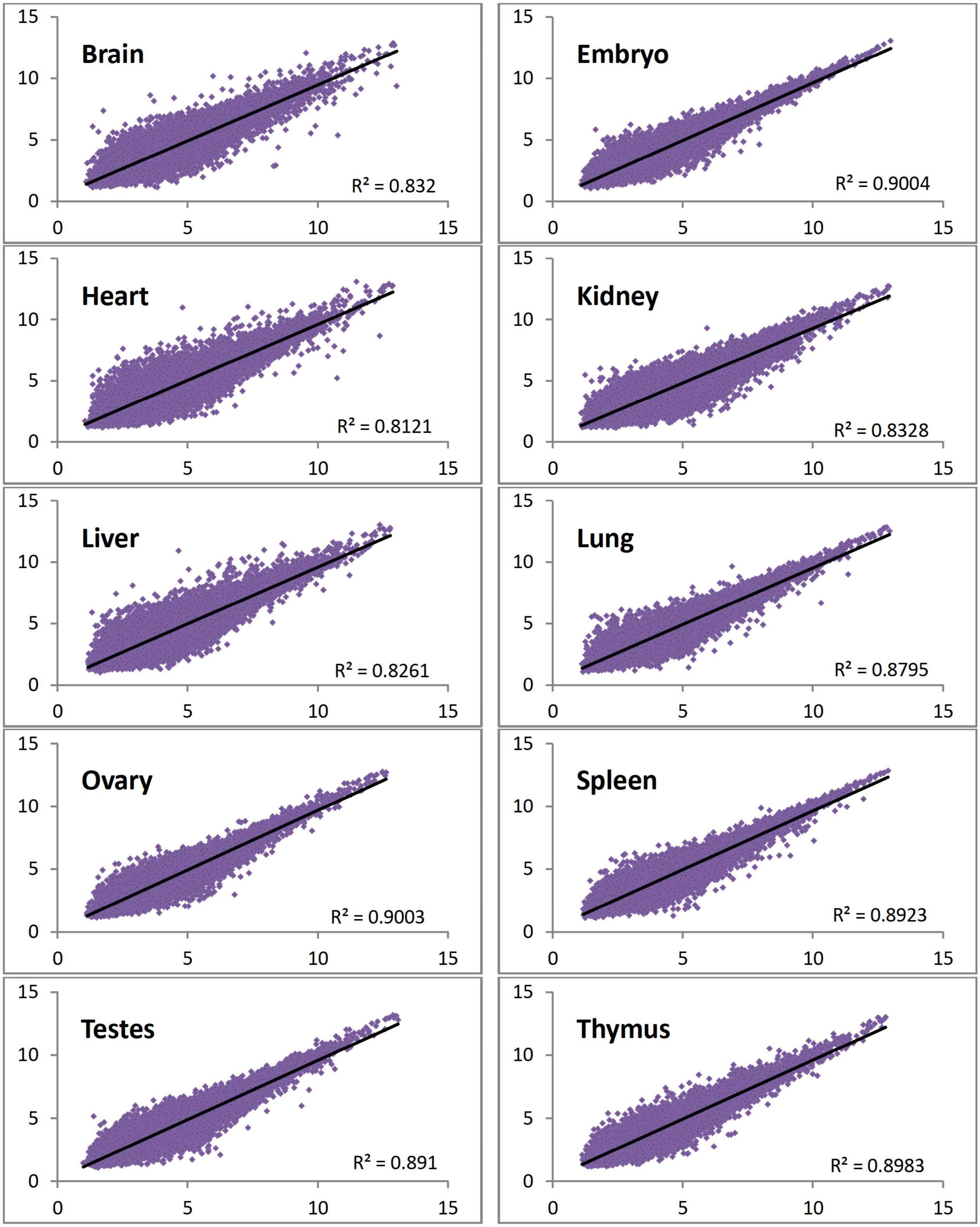

Figure S20. Correlation between intensity of technical replicates (n = 2) for mouse antisense exon array. The y-axis and x-axis are the expression intensities (in logarithmic scale) of the first and second replicate respectively. **Main Findings: All technical replicates are correlated.**

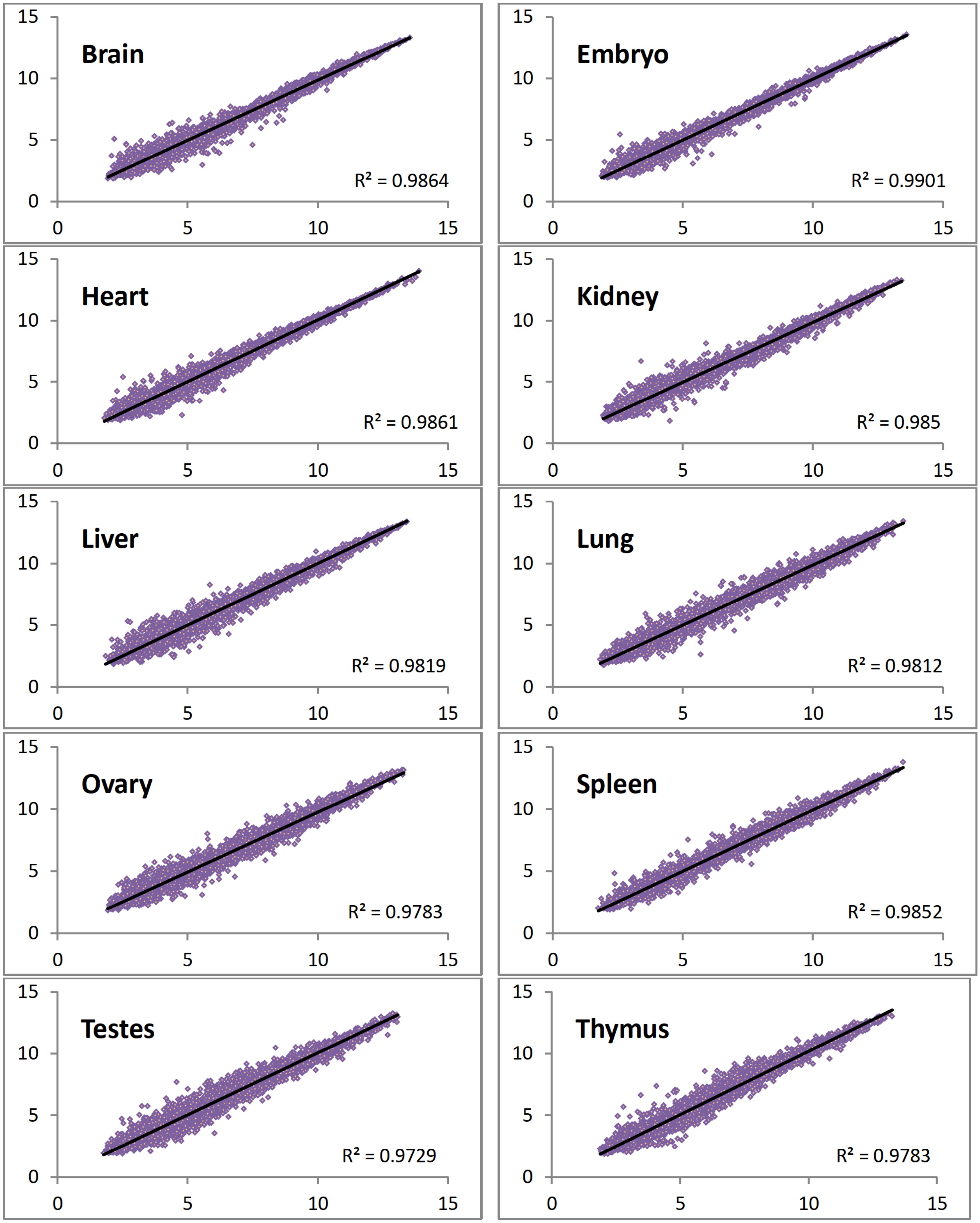

Figure S21. Correlation between intensity of technical replicates (n = 2) for rat sense gene array. The y-axis and x-axis are the expression intensities (in logarithmic scale) of the first and second replicate respectively. **Main Findings: All technical replicates are correlated.**

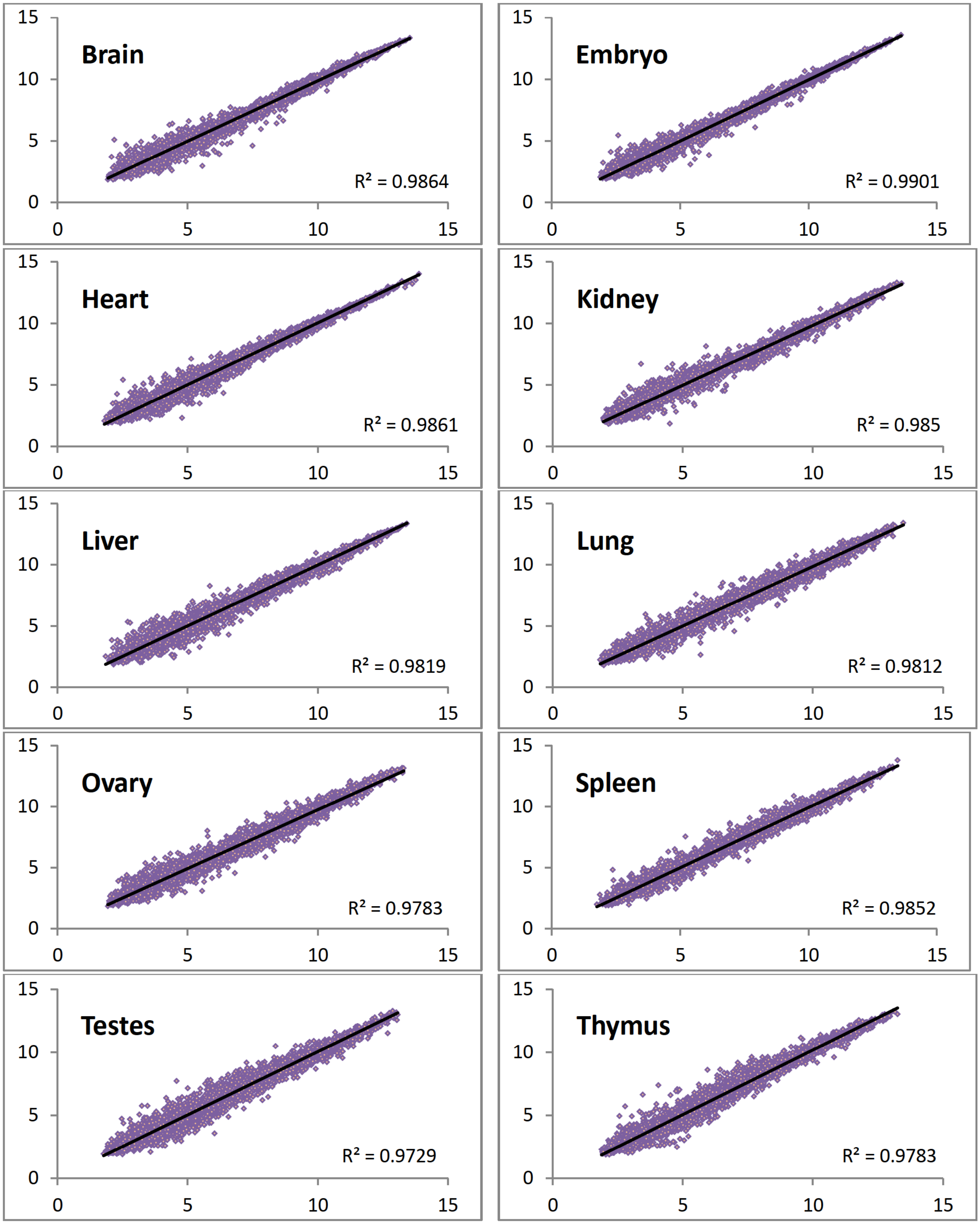

Figure S22. Correlation between intensity of technical replicates (n = 2) for rat antisense gene array. The y-axis and x-axis are the expression intensities (in logarithmic scale) of the first and second replicate respectively. **Main Findings: All technical replicates are correlated.**

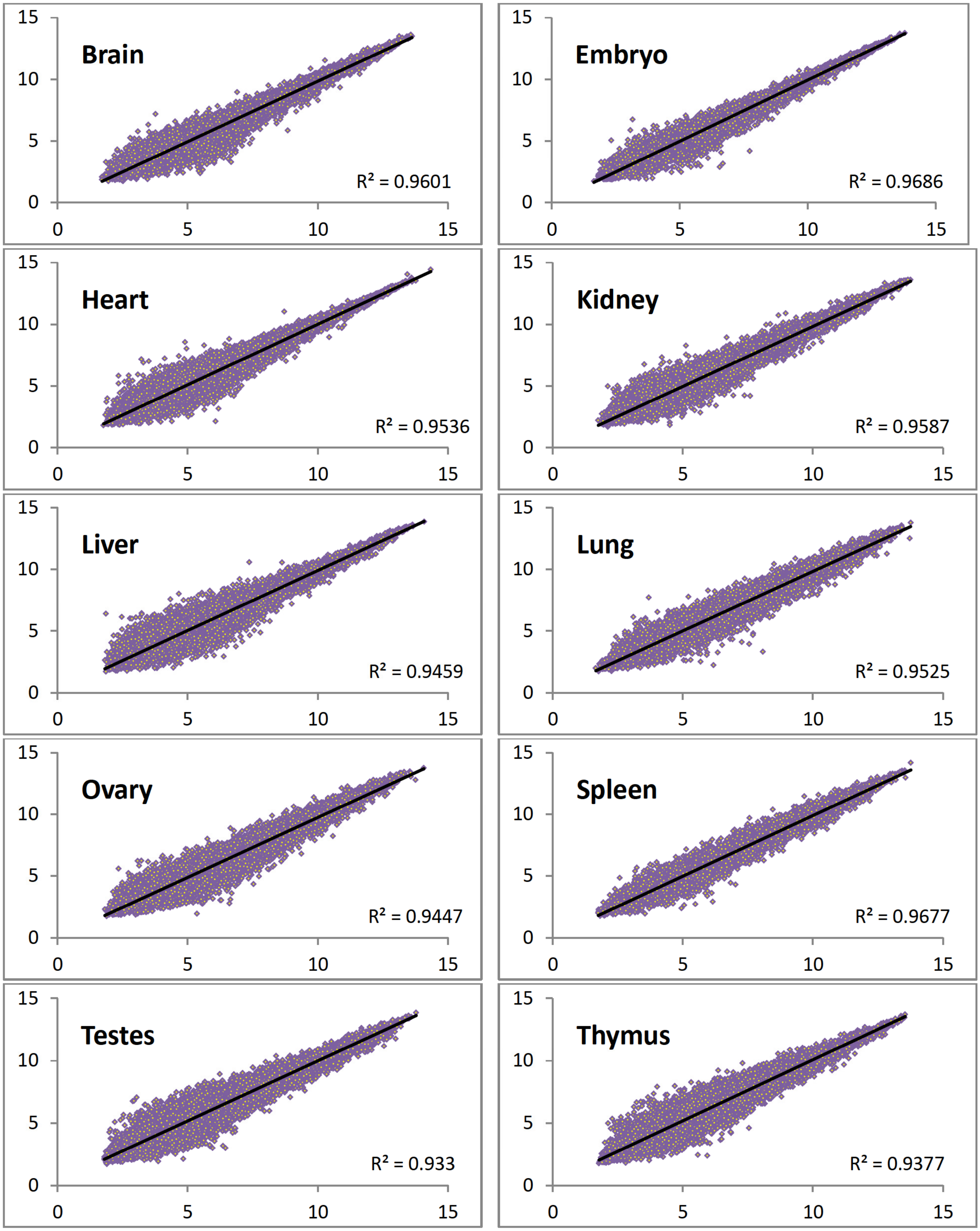

Figure S23. <u>Correlation between intensity of technical replicates (n = 2) for rat sense exon array.</u> The y-axis and x-axis are the expression intensities (in logarithmic scale) of the first and second replicate respectively. **Main Findings: All technical replicates are correlated.**

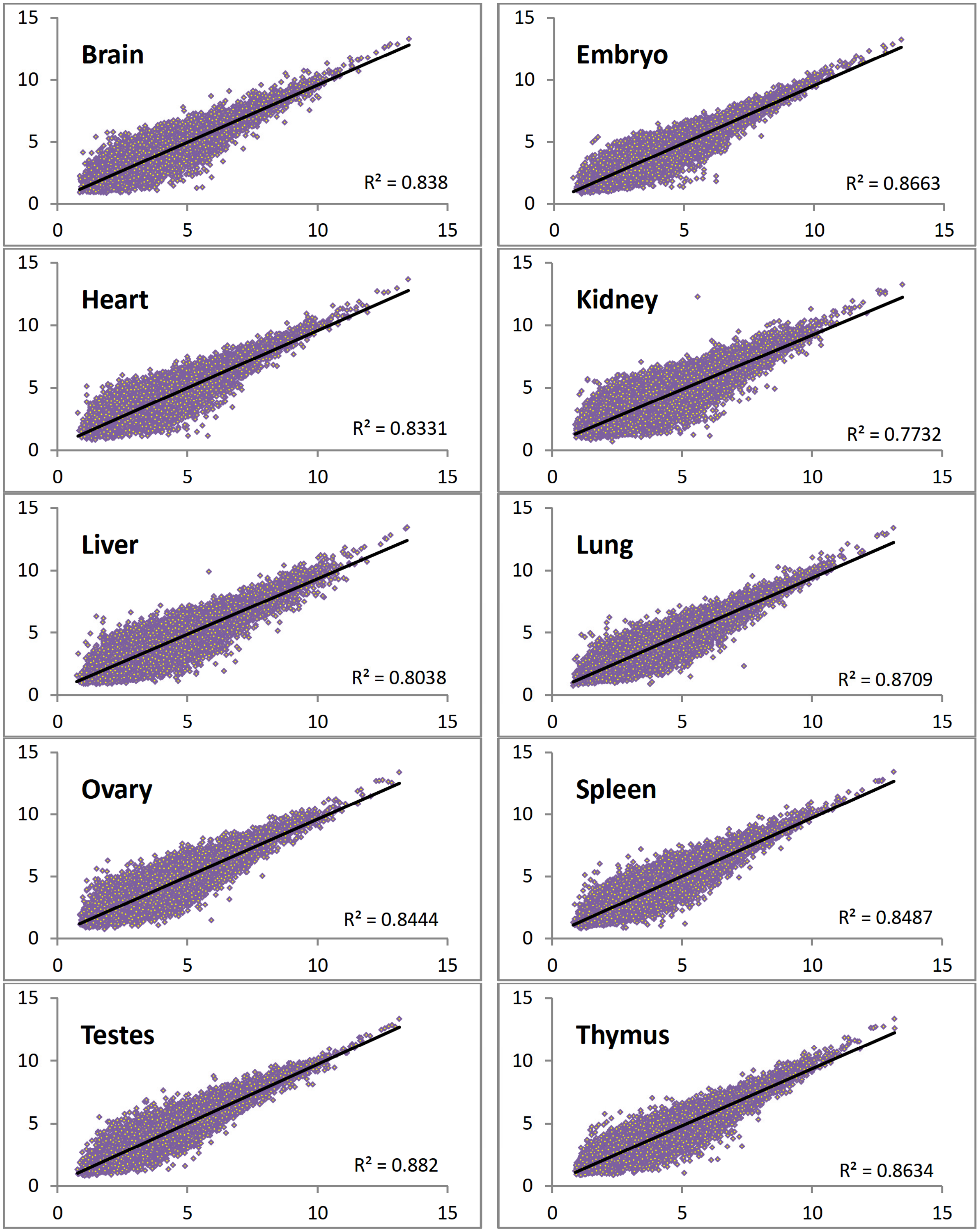

Figure S24. Correlation between intensity of technical replicates (n = 2) for rat antisense exon array. The y-axis and x-axis are the expression intensities (in logarithmic scale) of the first and second replicate respectively. **Main Findings: All technical replicates are correlated.**

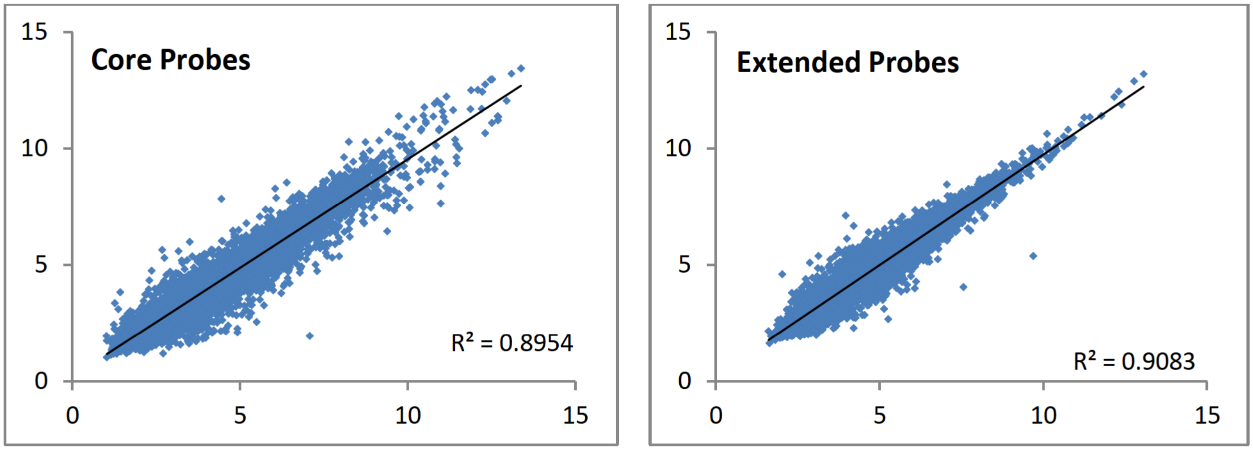

Figure S25. Comparing the correlations of the 2 technical replicates between overlapping core and extended probesets for human brain. 5283 extended probesets were found to be in opposite orientation compared to the core probesets with less than 25 bases between the centre of the opposite oriented probesets. This allowed for the validation of the antisense array protocol using the extended probesets in the corresponding sense array. Using these 5283 probesets, correlation between the technical replicates (n = 2) were estimated.

Main Findings: The technical replicates are strongly correlated in both core probesets in antisense array and extended probesets in sense array.

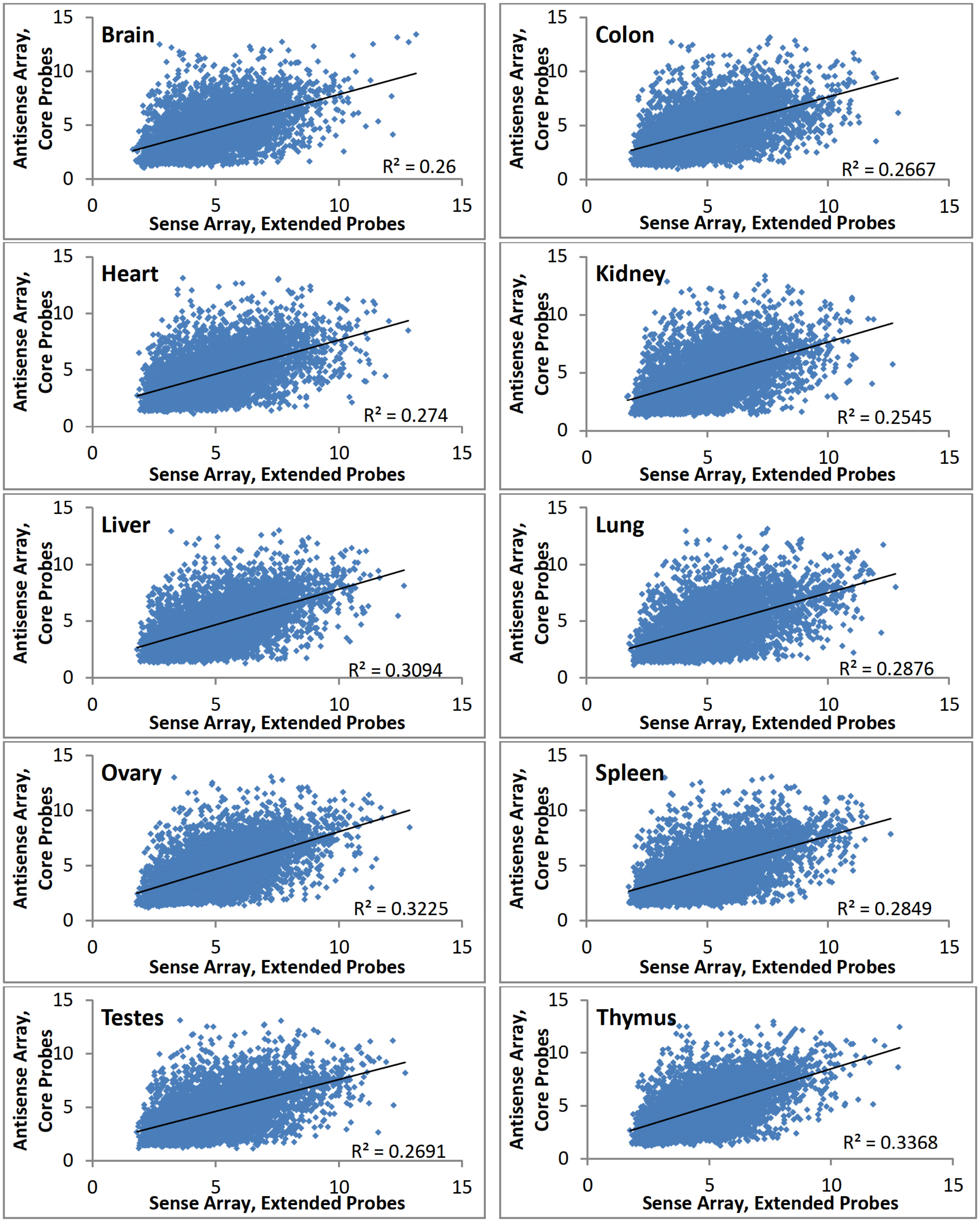

Figure S26. <u>Correlation between overlapping core probesets and oppositely oriented extended probesets in human arrays.</u> 5283 extended probesets were found to be in opposite orientation compared to the core probesets with less than 25 bases between the centre of the opposite oriented probesets. This allowed for the validation of the antisense array protocol using the extended probesets

in the corresponding sense array. The expression intensity of each probeset is the average of the 2 technical replicates. **Main Findings: Using 10 human tissues, the correlation between the overlapping core probesets and the oppositely oriented extended probesets is between 0.25 to 0.34.**

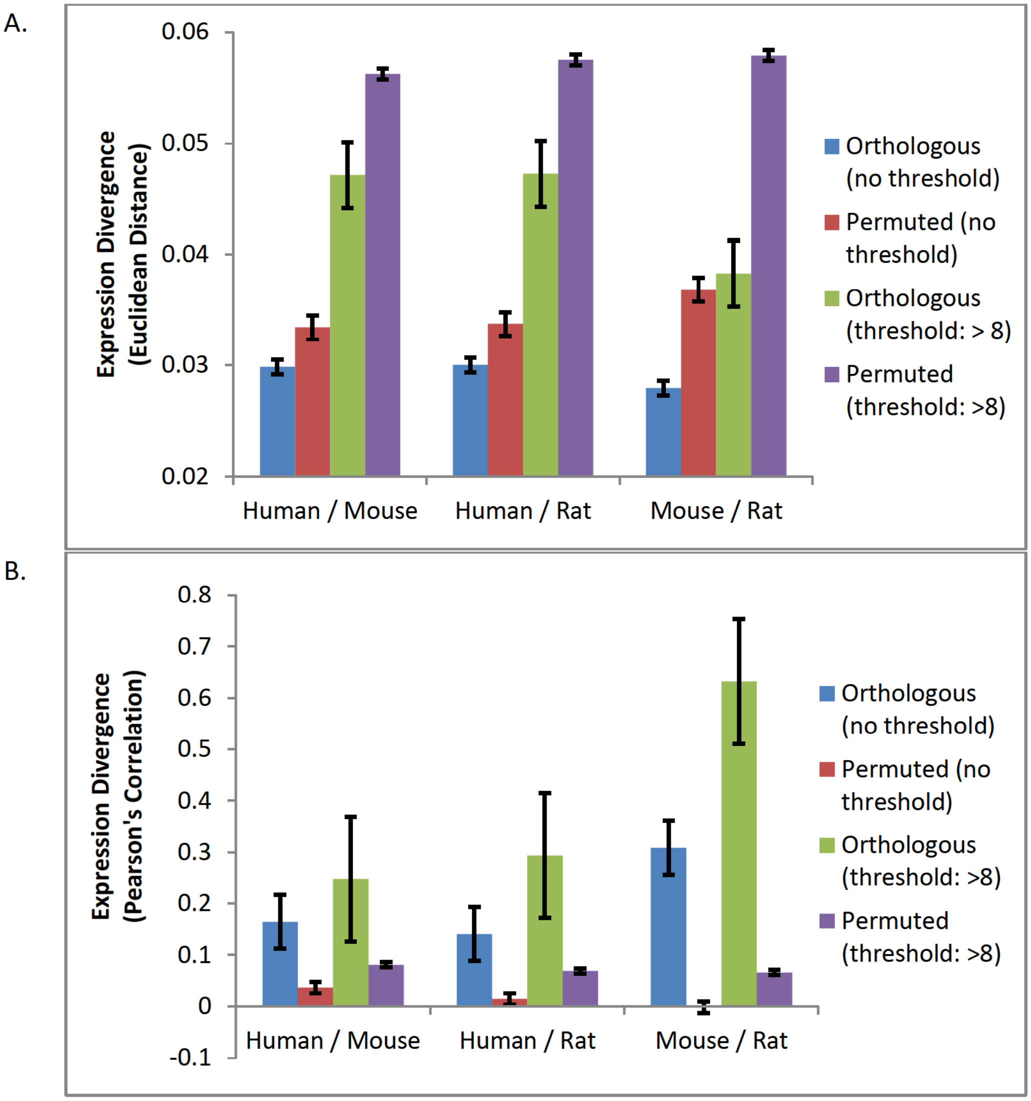

Figure S27. Average expression divergence between orthologous sense genes and permuted pairs. Panel A used Euclidean distance as a measure of expression divergence while Panel B used Pearson's correlation. "No threshold" refers to using full set of orthologous genes. "Threshold: >8" requires at least one of the orthologous probesets to have an expression intensity of more than 8, so as to reduce errors due to lowly expressed genes in both species. Error bars denote standard error.

Main Findings: Orthologous pairs show expression divergence which are significantly lower than that of permuted pairs regardless of threshold or measures, suggesting that different threshold or measures do not affect the conclusion. Mouse/rat orthologous genes show lower expression divergence compared to either human/mouse or human/rat orthologous genes. These findings are consistent with Jordan et al. (2005).

A.

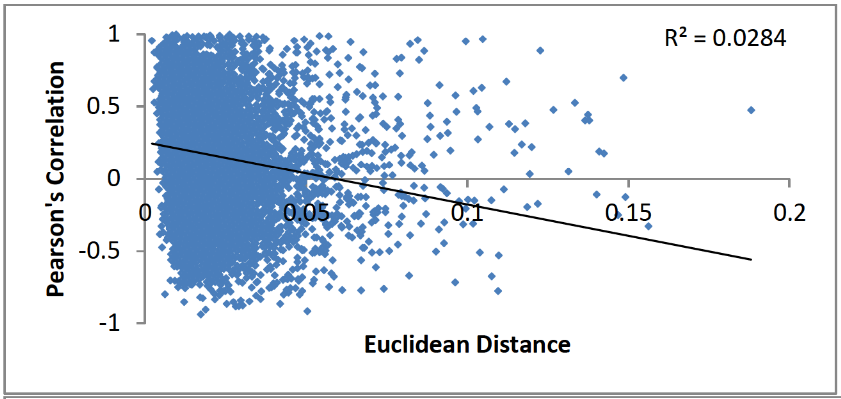

B.

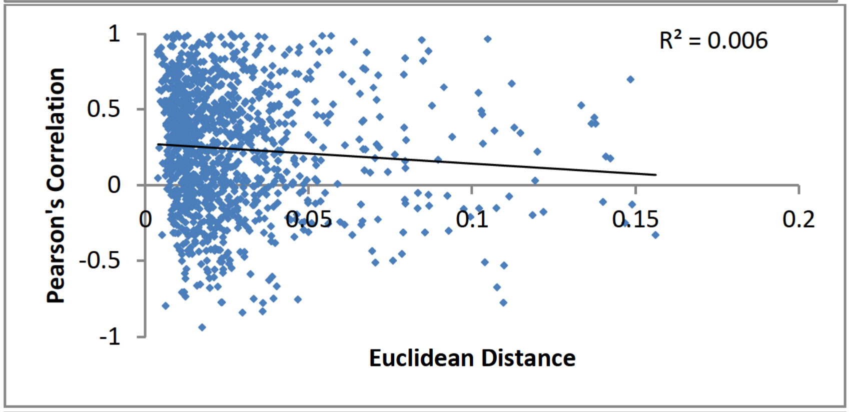

C.

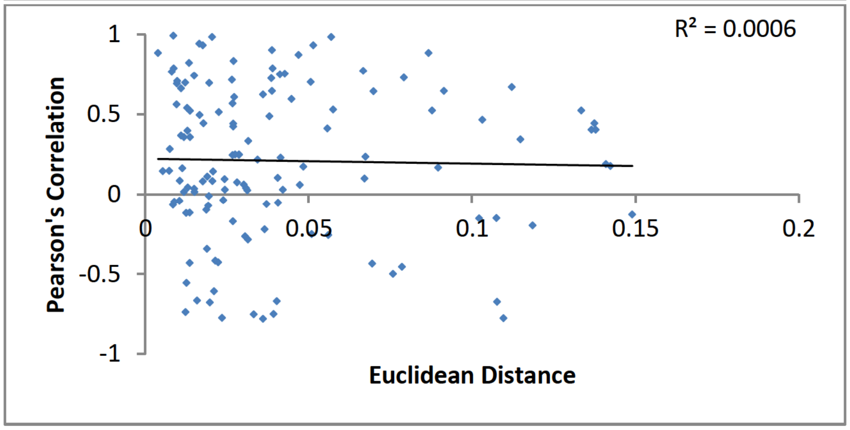

Figure S28. <u>Correlation between Euclidean distance and Pearson's Correlation.</u> The correlation between Euclidean distance and Pearson's Correlation was analyzed using orthologous sense gene expressions between human and mouse. Panel A shows the scatterplot using all orthologous gene expressions between human and mouse while Panels B and C requires at least one of the expressions in the orthologous tissues to be more than 6.5 and 8 respectively. **Main Findings: There is no correlation between Euclidean distance and Pearson's correlation.**

A.

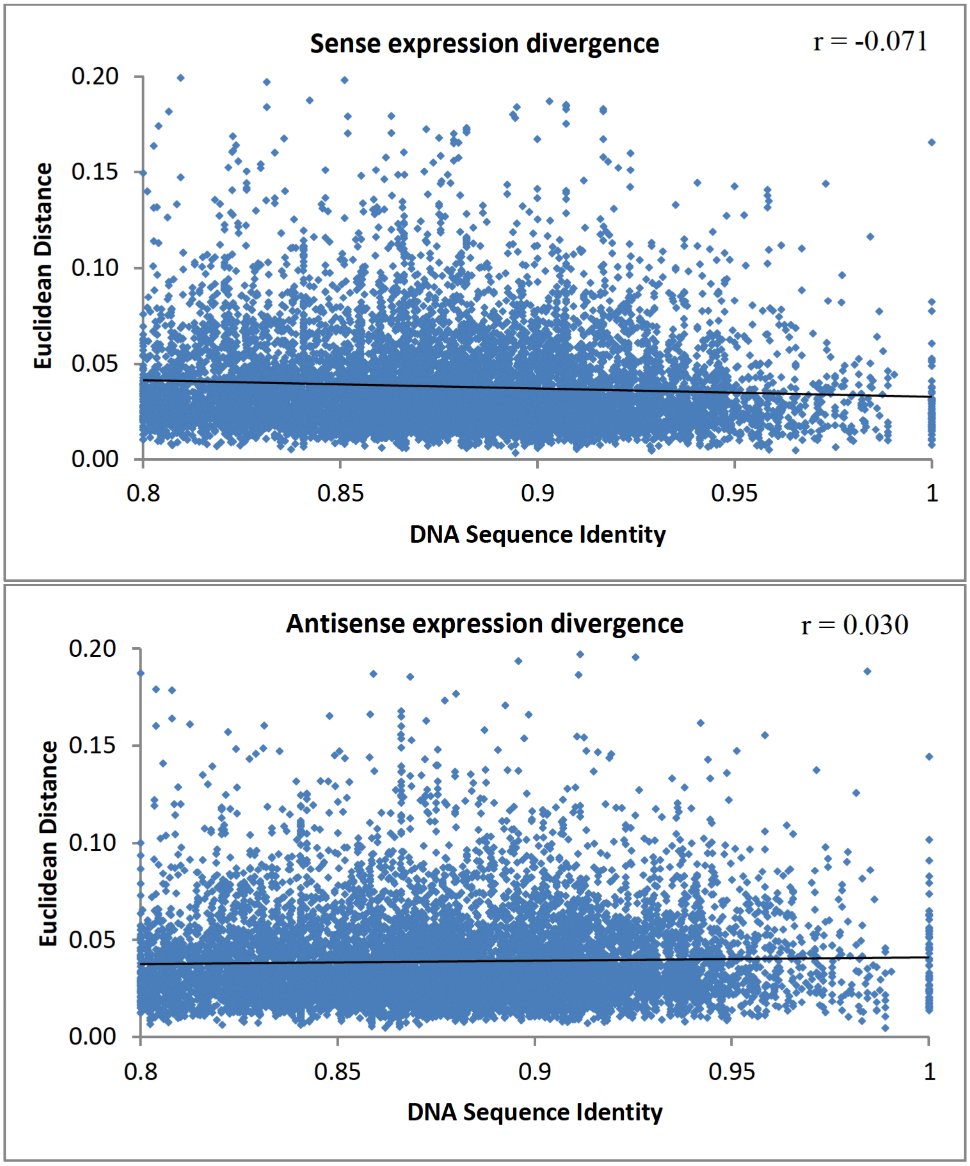

Figure S29. <u>Correlation between expression divergence and DNA sequence identity.</u>

Main Findings: Expression divergence is not correlated to DNA sequence identity for both sense and antisense transcripts.

A.

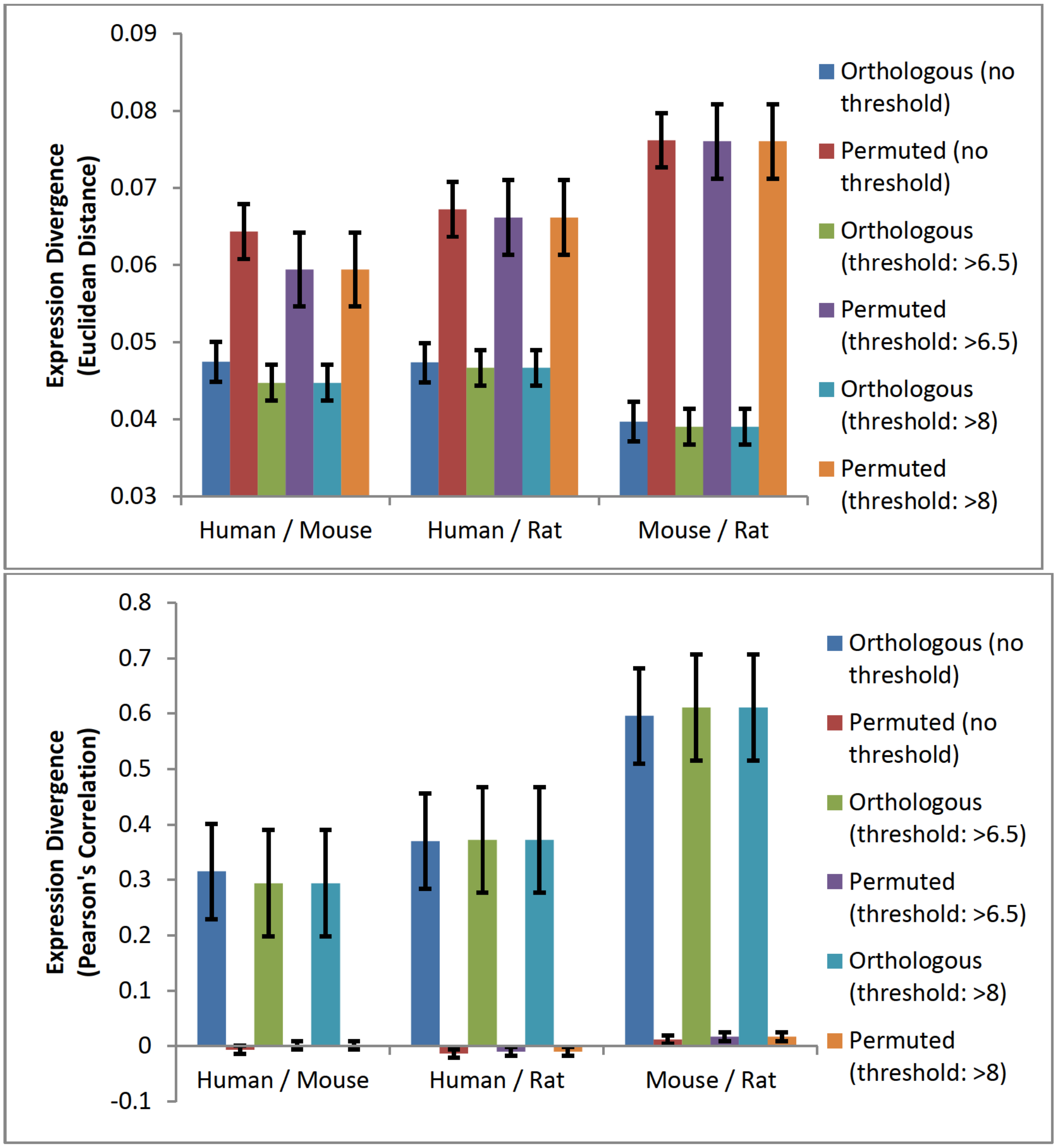

Figure S30. <u>Average expression divergence between orthologous sense exon probesets and permuted pairs.</u> Panel A used Euclidean distance as a measure of expression divergence while Panel B used Pearson's correlation. "No threshold" refers to using full set of orthologous genes. "Threshold: >6.5" and "Threshold: >8" requires at least one of the orthologous probesets to have an expression intensity of more than 6.5 and 8 respectively. Error bars denote standard error.

Main Findings: Orthologous pairs show expression divergence which are significantly lower than that of permuted pairs regardless of threshold or measures, suggesting that different threshold or measures do not affect the conclusion. Mouse/rat orthologous genes show lower expression divergence compared to either human/mouse or human/rat orthologous genes. These findings are consistent with Jordan et al. (2005).

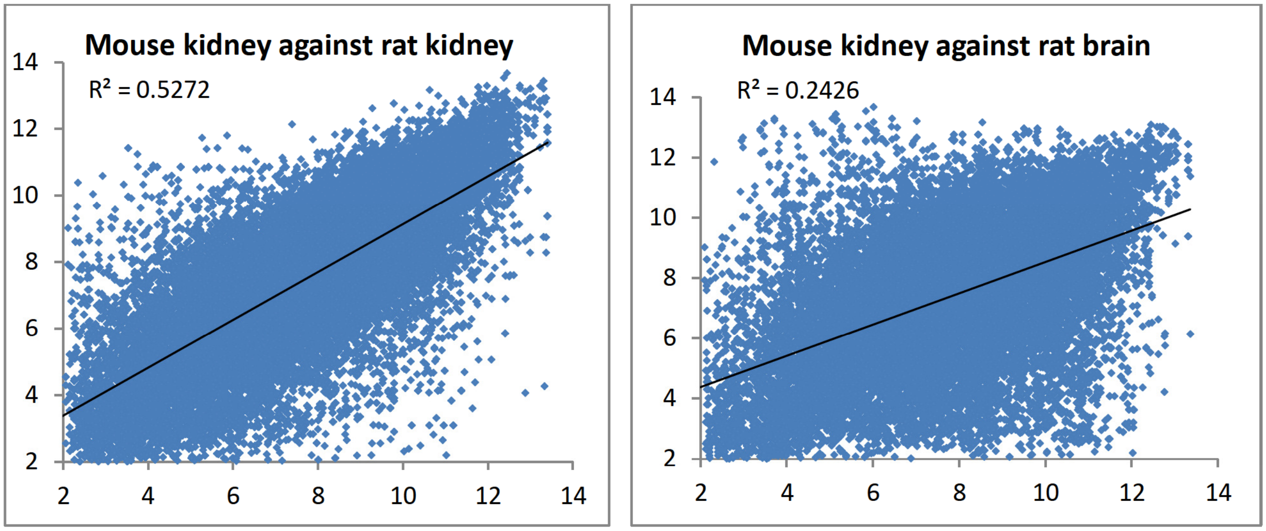

Figure S31. Scatterplot between sense exon expression of mouse kidney and rat kidney or rat brain. Both axes are expression levels. **Main Findings: Orthologous tissues are more correlated in expression levels than non-orthologous tissues for sense expression.**

A.

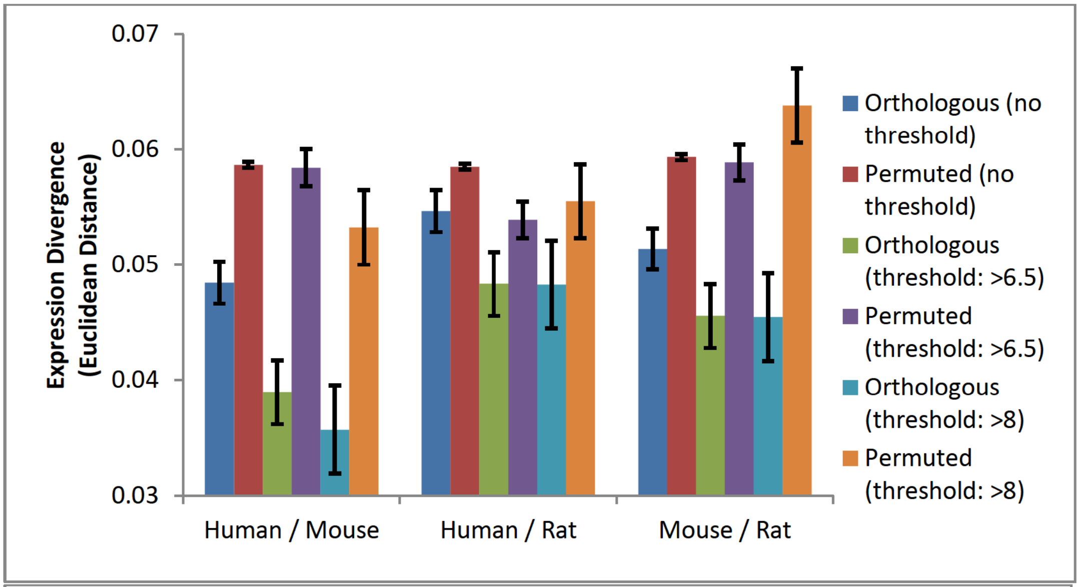

B.

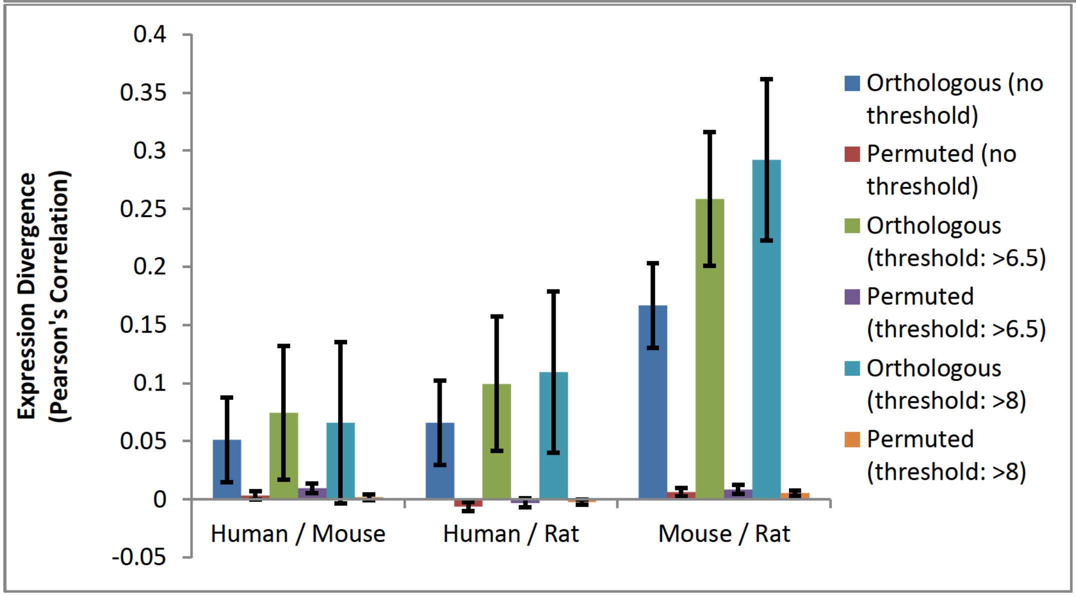

Figure S32. Average expression divergence between orthologous antisense transcript probesets at exon level and permuted pairs (with overlapping RefSeq transcripts as antisense transcripts). Panel A used Euclidean distance as a measure of expression divergence while Panel B used Pearson's correlation. "No threshold" refers to using full set of orthologous genes. "Threshold: >6.5" and "Threshold: >8" requires at least one of the orthologous probesets to have an expression intensity of more than 6.5 and 8 respectively. Error bars denote standard error.

Main Findings: Orthologous pairs show expression divergence which are significantly lower than that of permuted pairs regardless of threshold or measures, suggesting that different threshold or measures do not affect the conclusion.

A.

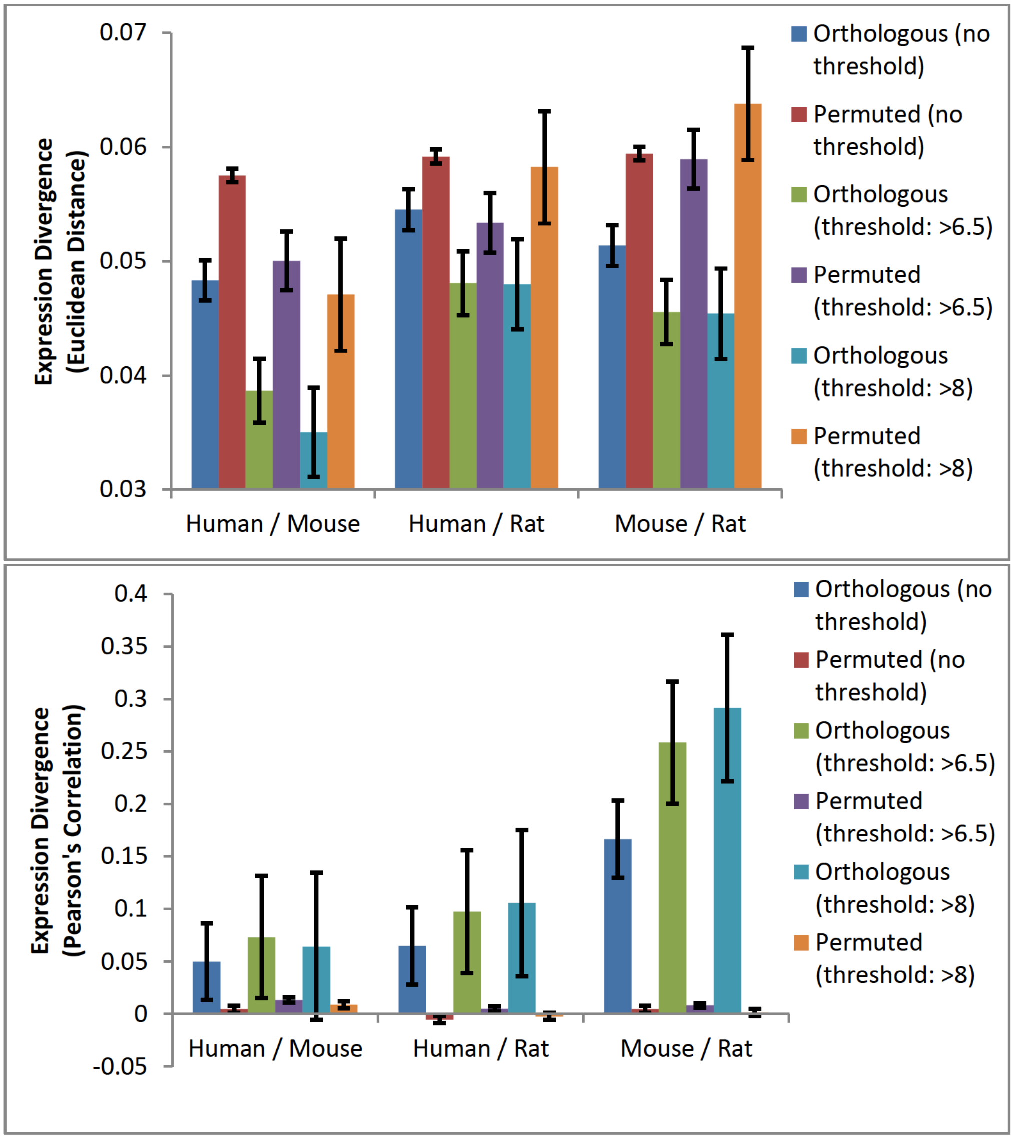

Figure S33. <u>Average expression divergence between orthologous antisense transcript probesets at exon level and permuted pairs (after removal of overlapping RefSeq transcripts as antisense transcripts).</u> Panel A used Euclidean distance as a measure of expression divergence while Panel B used Pearson's correlation. "No threshold" refers to using full set of orthologous genes. "Threshold: >6.5" and "Threshold: >8" requires at least one of the orthologous probesets to have an expression intensity of more than 6.5 and 8 respectively. Error bars denote standard error.

Main Findings: Orthologous pairs show expression divergence which are significantly lower than that of permuted pairs regardless of threshold or measures, suggesting that different threshold or measures do not affect the conclusion. Comparing to Figure S26, the presence of interfering RefSeq transcripts from the opposite strand does not affect the conclusion.

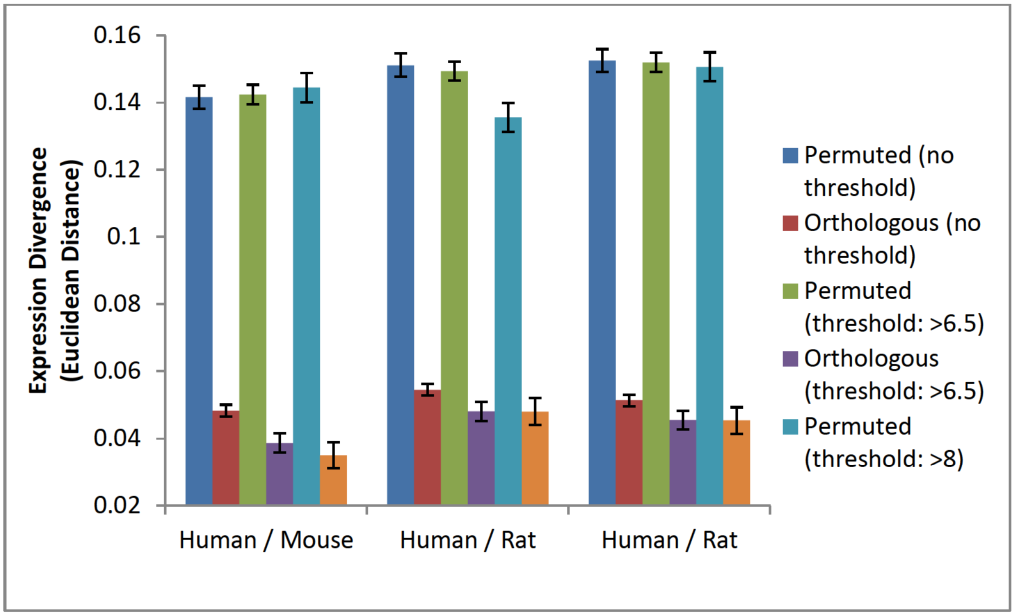

Figure S34. Average expression divergence between orthologous antisense transcript probesets at exon level and permuted pairs using Piasecka et al. (2012) randomization procedure to generate permuted pairs (after removal of overlapping RefSeq transcripts as antisense transcripts). “No threshold” refers to using full set of orthologous genes. “Threshold: >6.5” and “Threshold: >8” requires at least one of the orthologous probesets to have an expression intensity of more than 6.5 and 8 respectively. Error bars denote standard error.

Main Findings: Orthologous pairs show expression divergence which are significantly lower than that of permuted pairs regardless of threshold or measures, suggesting that different threshold or measures do not affect the conclusion. Comparing to Figure S26, the presence of interfering RefSeq transcripts from the opposite strand does not affect the conclusion.

A.

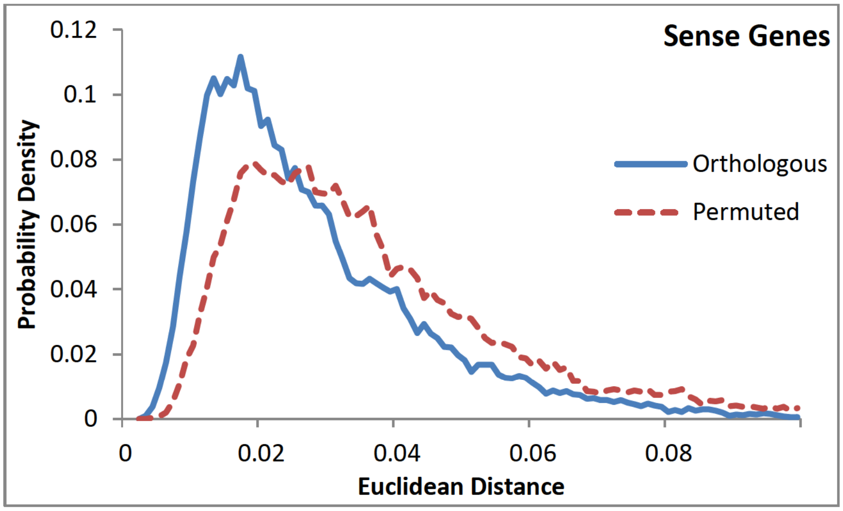

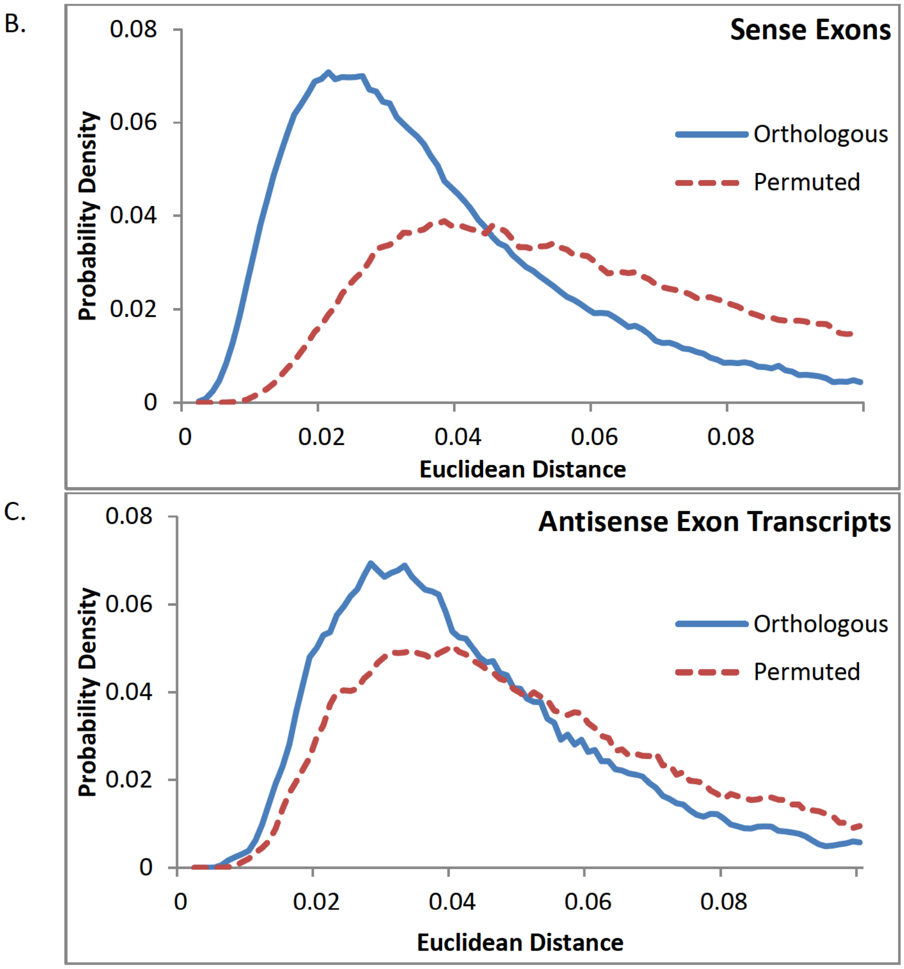

Figure S35. Distribution of mouse/rat orthologous transcripts at gene and exon level again permuted pairs. Vertical axis represents cumulative frequency. Horizontal axis represents expression divergence, calculated using Euclidean distance. Panel A shows the expression divergence distribution of protein-coding genes as defined by NCBI HomoloGene build 65 and its permuted pairs. Panel B shows the orthologous sense exons. Panel C shows antisense exon transcripts are the exon level which excluded exons with antisense RefSeq transcripts.

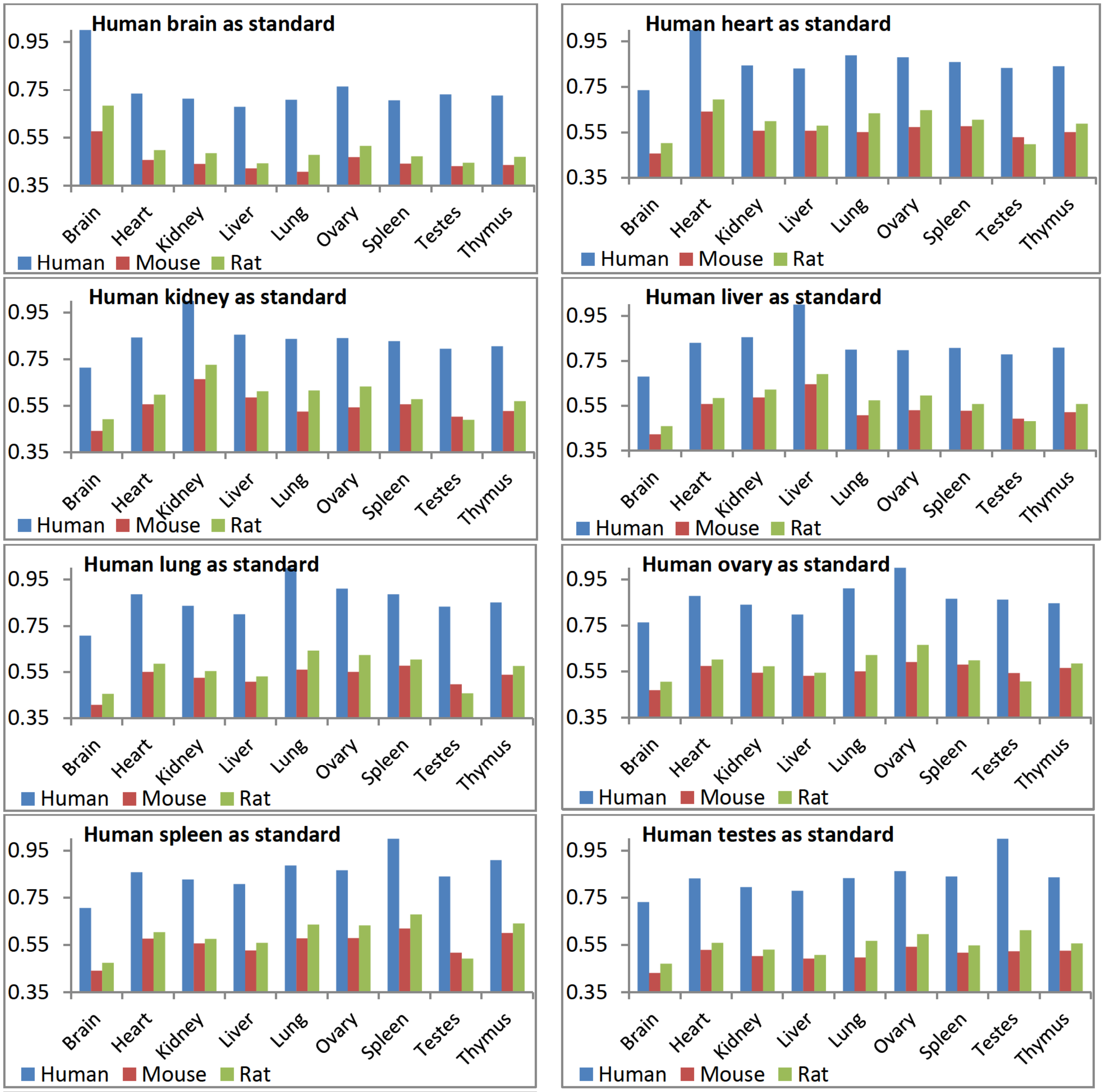

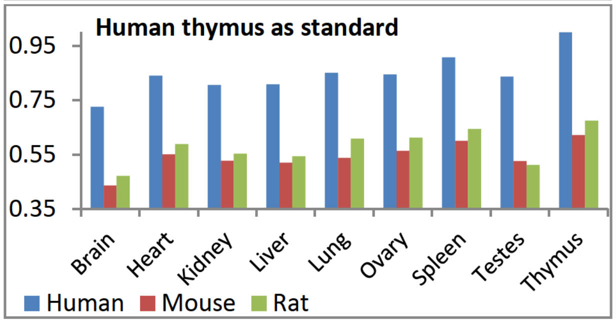

Figure S36. <u>Expression correlations between all tissues using human tissues as standards (sense exon array).</u> The y-axis represents Pearson's correlation between the tissue used for standard and other tissues of the same organism and orthologous tissues in mouse and rat. **Main Findings: Higher correlation in human tissues compared with mouse or rat tissues. Among rat and mouse tissues, the orthologous tissues are more correlated than the rest.**

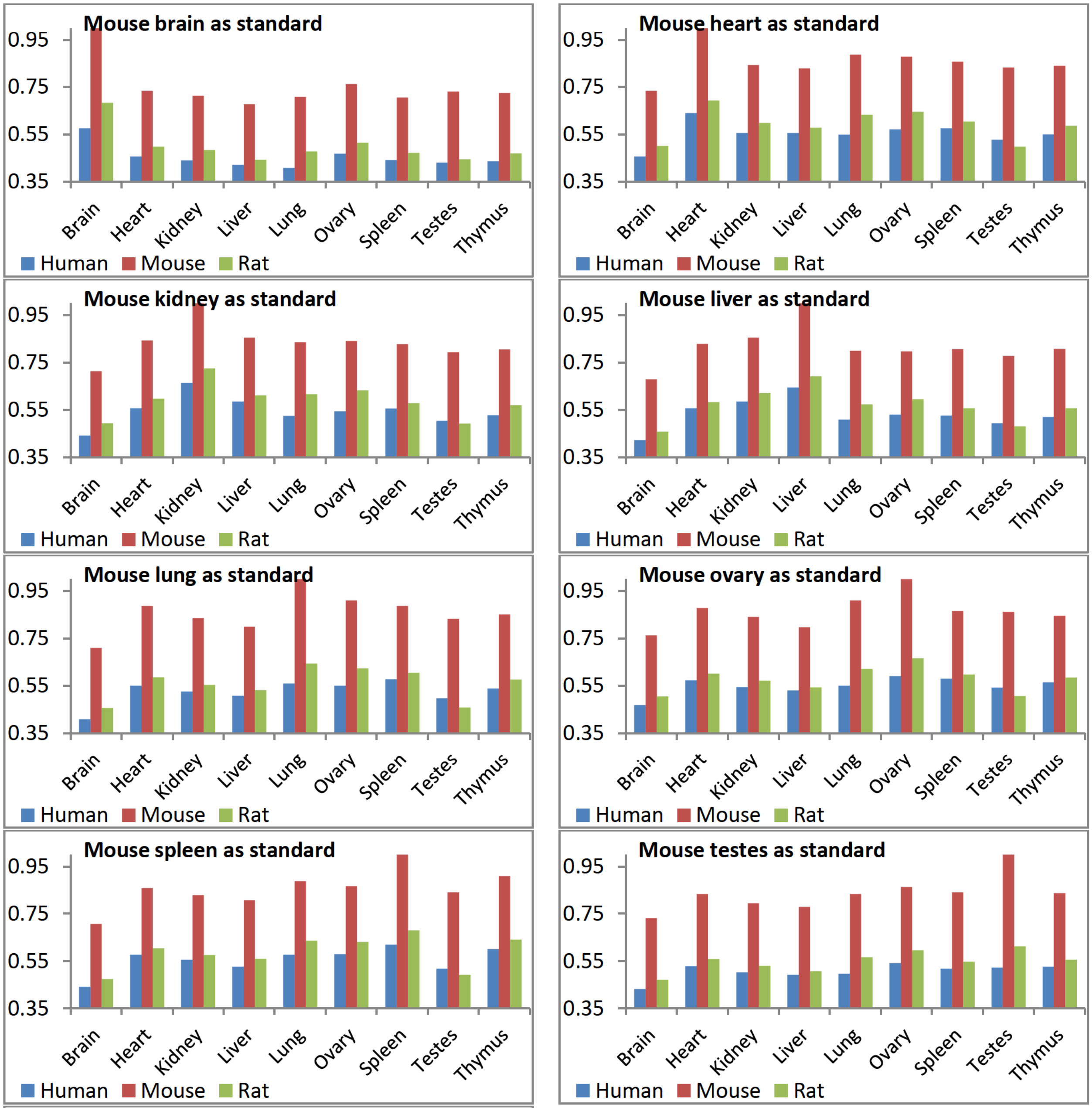

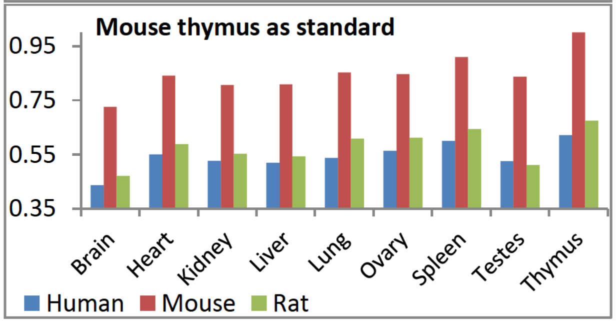

Figure S37. Expression correlations between all tissues using mouse tissues as standards (sense exon array). The y-axis represents Pearson's correlation between the tissue used for standard and other tissues of the same organism and orthologous tissues in human and rat. **Main Findings: Higher correlation in mouse tissues compared with human or rat tissues. Among rat and human tissues, the orthologous tissues are more correlated than the rest.**

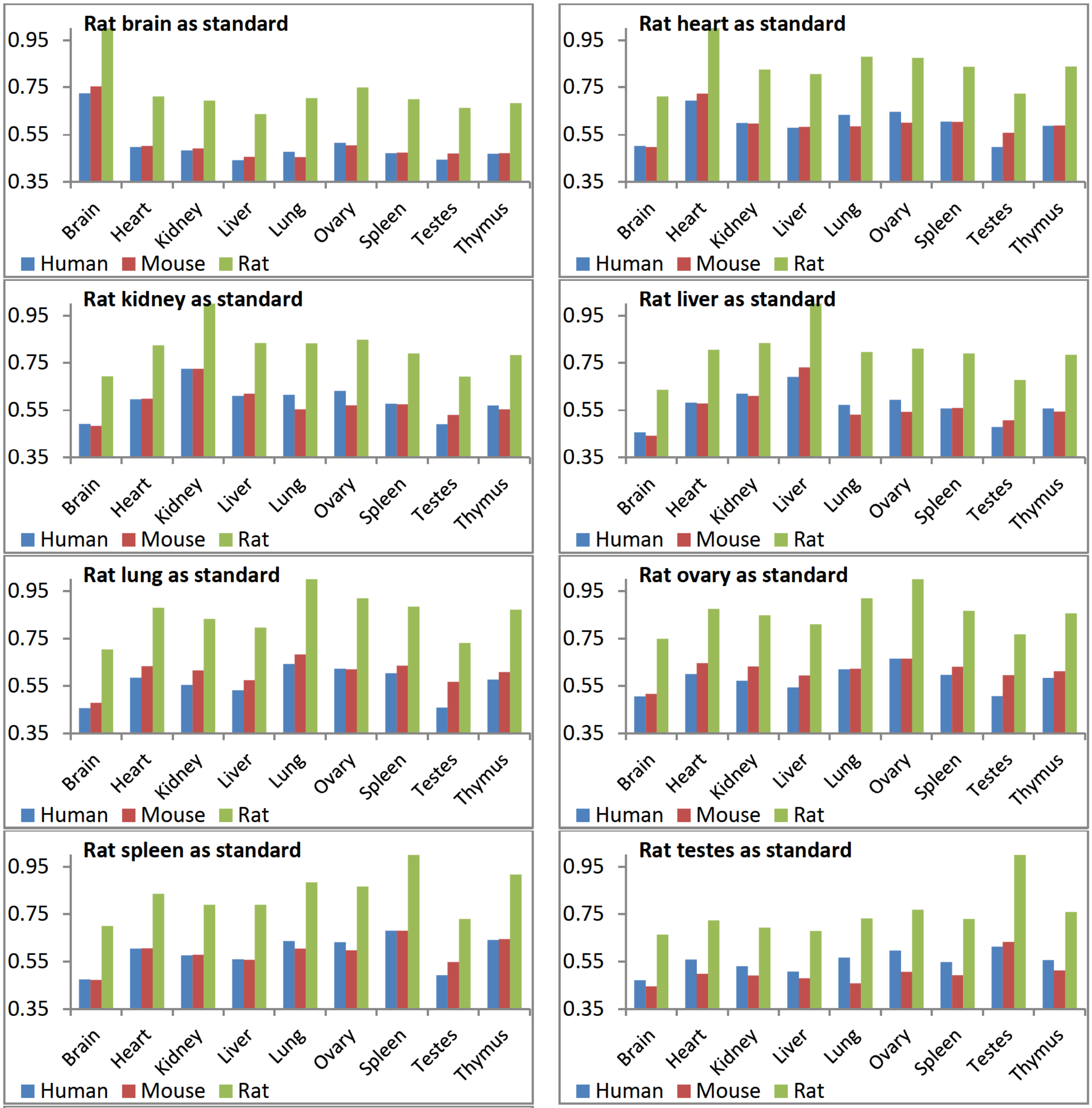

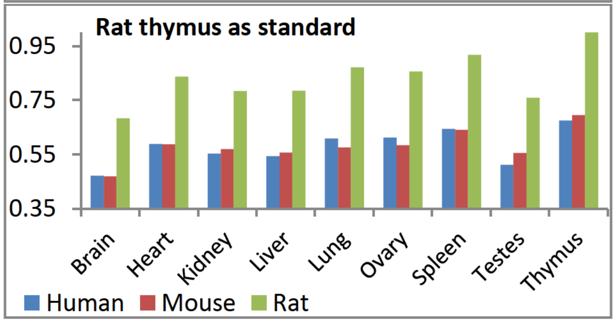

Figure S38. Expression correlations between all tissues using rat tissues as standards (sense exon array). The y-axis represents Pearson's correlation between the tissue used for standard and other tissues of the same organism and orthologous tissues in human and mouse. **Main Findings: Higher correlation in rat tissues compared with human or mouse tissues. Among mouse and human tissues, the orthologous tissues are more correlated than the rest.**

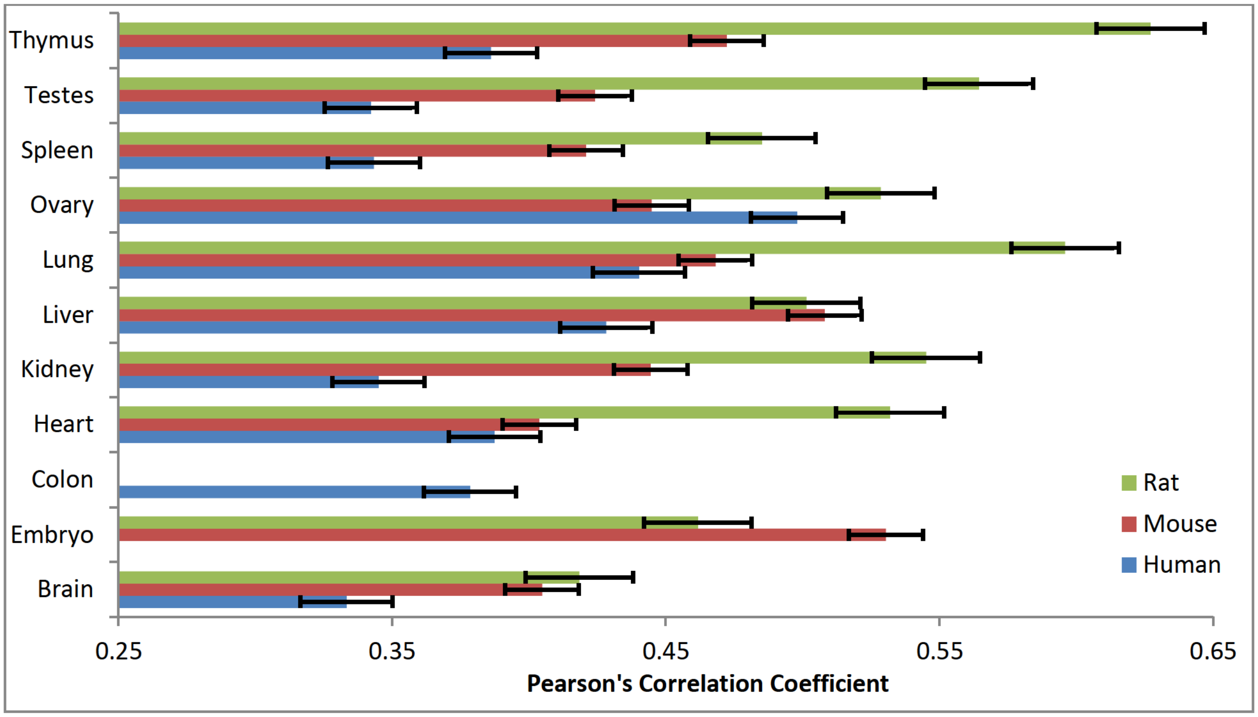

Figure S39. Correlation between sense and antisense expression levels. Error bars represent standard error.

Code S1: R code for Piasecka et al. (2012) randomization

```
##################################################################################
# randomization process according to Piasecka et al. (Bioinformatics 2012)#
##################################################################################
# PART I. Functions #
######################
# TAU calculation function
TAU_calc = function(DAT){
	X_head = t(apply(DAT,1,function(h)h/max(h)))
	N = ncol(DAT)
	apply(X_head, 1, function(h)sum(1-h)/(N-1))
}

# randomization function according to uniform TAU
Uniform_tau_rand = function(DAT, N){
	N_rand = runif(N, min=min(DAT), max=max(DAT))
	INDEX = rep(0, N)
	for(i in 1:N){
		INDEX[i] = which.min(abs(DAT-N_rand[i]))[1]
	}
	INDEX
}

# expression divergence. relative abundance
eura2 = function(data1, data2){
	Vec_d = rep(0,nrow(data1))
	for (i in 1:nrow(data1)){
		expr.1 = as.numeric(as.matrix(data1[i,]))
		expr.2 = as.numeric(as.matrix(data2[i,]))
		expr.1 = expr.1/sum(expr.1)
		expr.2 = expr.2/sum(expr.2)
		d = sqrt(sum((expr.1-expr.2)^2))
		if(is.na(d)){
			Vec_d[i]=NA
		}else{
			Vec_d[i]=d
		}
	}
	Vec_d
}

#################
# PART II. Main #
#################
main_function = function(N, sourcefile, h_outputfile, r_outputfile){
	# READ data
	DATA = read.csv(sourcefile)
```

```
        # split data with labels "1" and "2" representing the 2 sets of tissues
        DATA_s1 = DATA[,grep("1",names(DATA),value=T)]
        DATA_s2 = DATA[,grep("2",names(DATA),value=T)]

        # calculate TAU
        TAU_s1 = TAU_calc(DATA_s1)
        TAU_s2 = TAU_calc(DATA_s2)

        # randomized uniform TAU with N, result is a vector of
        #   randomized indexs corresponding to gene expression data
        Index_rand_s1 = Uniform_tau_rand(TAU_s1, N)
        Index_rand_s2 = Uniform_tau_rand(TAU_s2, N)

        # calculate expression divergence, result is a vector
        # empirical ED
        ExprDiv_empirical = eura2(DATA_s1, DATA_s2)

        # ED calculated from randomized data
        ExprDiv_rand = eura2(DATA_s1[Index_rand_s1, ], DATA_s2[Index_rand_s2, ])

        # write homologous and randomized EDs into files
    write.table(ExprDiv_rand, r_outputfile, row.names=F,col.names=F,quote=F,sep="\t")
    write.table(ExprDiv_empirical, h_outputfile, row.names=F,col.names=F,quote=F,sep="\t")
}

# main_function(<number of randomized pairs>,
#           <Orthologous sample file (CSV)>)
#           <Orthologous pairs ED filename (for output)>)
#           <Randomized pairs ED filename (for output)>)
main_function(30000, "Human-Mouse-Antisense-Exon.csv", "homologous.txt",  "random.txt")
```
